# Rapid Fabrication of Low-Cost Thermal Bubble-Driven Micro-Pumps

**DOI:** 10.3390/mi13101634

**Published:** 2022-09-29

**Authors:** Brandon Hayes, Lawrence Smith, Heiko Kabutz, Austin C. Hayes, Gregory L. Whiting, Kaushik Jayaram, Robert MacCurdy

**Affiliations:** Paul M. Rady Department of Mechanical Engineering, University of Colorado Boulder, Boulder, CO 80309, USA

**Keywords:** bubble dynamics, microfluidics, low-cost, phase change, inertial pumping

## Abstract

Thermal bubble-driven micro-pumps are an upcoming actuation technology that can be directly integrated into micro/mesofluidic channels to displace fluid without any moving parts. These pumps consist of high power micro-resistors, which we term thermal micro-pump (TMP) resistors, that locally boil fluid at the resistor surface in microseconds creating a vapor bubble to perform mechanical work. Conventional fabrication approaches of thermal bubble-driven micro-pumps and associated microfluidics have utilized semiconductor micro-fabrication techniques requiring expensive tooling with long turn around times on the order of weeks to months. In this study, we present a low-cost approach to rapidly fabricate and test thermal bubble-driven micro-pumps with associated microfluidics utilizing commercial substrates (indium tin oxide, ITO, and fluorine doped tin oxide, FTO, coated glass) and tooling (laser cutter). The presented fabrication approach greatly reduces the turn around time from weeks/months for conventional micro-fabrication to a matter of hours/days allowing acceleration of thermal bubble-driven micro-pump research and development (R&D) learning cycles.

## 1. Introduction

Thermal bubble-driven micro-pumps (also known as inertial micro-pumps) are an upcoming actuation technology for moving fluid without the use of external pump sources [1,2,3,4,5]. These devices use a small thin film heater to rapidly boil liquid, creating a vapor bubble that displaces adjacent fluid. The ability to directly integrate these micro-pumps into micro/mesofluidic channels eliminates the need for large, bulky external syringe pumps or pressure sources as is common in most commercial micro/mesofluidic systems thus enabling “lab-on-a-chip” technologies [6]. Although integrated pneumatic valves and pumps have been demonstrated and been highly successful in microfluidic technology [7,8], such systems necessitate the use of external pressure sources which increases the overall size of lab-on-a-chip devices. As such, thermal bubble-driven micro-pumps show great promise in their simplicity and direct integration without the need for external flow or pressure sources. Yet, thermal bubble-driven micro-pump development is still in its infancy; moreover, R&D learning cycles are often long (weeks or months) due to the use of semiconductor micro-fabrication techniques [9,10]. The reliance on semiconductor micro-fabrication techniques, albeit preferred in mass production, hinders this technology’s widespread accessibility to biomedical researchers and speed of development. Modeling off of the “fail fast and fail often” adage of the microfluidic community [11,12], the present work details a low-cost approach to rapidly fabricate and test thermal bubble-driven micro-pumps in a matter of hours/days instead of weeks/months. We envision this low-cost workflow as a means to enable both widespread accessibility of this technology as well as a means to rapidly iterate through initial R&D learning cycles to accelerate development of thermal bubble-driven micro-pumps for lab-on-a-chip systems.

Physically, thermal bubble-driven micro-pumps are high power micro-resistors, which we term thermal micro-pump (TMP) resistors, located inside a micro/mesofluidic channel. They were first theorized and demonstrated by Prosperetti et al. [13,14] in 2000 and commercialized by Hewlett-Packard [5] in the 2010’s. A voltage pulse lasting a few microseconds is applied generating a heat flux in excess of 500 W/mm${}^{2}$ that vaporizes a thin layer of fluid above the resistor’s surface creating a vapor bubble which displaces fluid and performs mechanical work [15]. When placed asymmetrically in a channel with reservoirs at either end, a momentum imbalance upon collapse results in a net fluid pumping effect [5,13,16,17,18]. Beyond simple pumping, thermal bubble-driven micro-pumps are successfully used as micro-mixers [19], fluid jets [20], fluid sorters/routers [4], and have been used for decades in commercial inkjet printers [21]. Yet, to enable commercial lab-on-a-chip systems, thermal bubble-driven micro-pumps must be studied with respect to biomedical applications. To date, TMP technology has been applied to cell lysis [22], sorting [23], and bioprinting [24]. However, little is known about the biocompatibility and stability of TMP resistors with blood and other biofluids, reagent stability when mixing with TMP resistors, and cell viability when pumping with TMP resistors. As such, there is a need for rapid prototyping of micro/mesofluidic devices with thermal bubble-driven micro-pumps to speed integration in lab-on-a-chip technologies.

**Figure 1:**
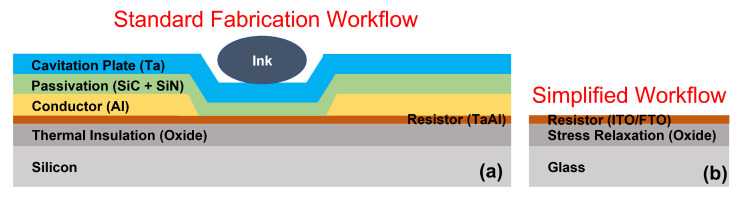
TMP Thin Film Stack—(**a**) illustrates the standard micro-fabrication thin film stack used in TMP resistors to heat ink which is based on thermal inkjet (TIJ) technology [21]. The stack is built on a silicon substrate followed by a thermal insulation layer, a resistive layer, a conductive layer, electrical passivation layers, and a cavitation plate. (**b**) shows the simplified commercial thin film stack used in this study for rapid fabrication of TMP resistors.

Thermal bubble-driven micro-pumps are currently fabricated almost exclusively using thin film micro-fabrication techniques [5,25,26,27,28,29,30]. In commercial TMP resistors, a special double-level metal interconnect integrated circuit (IC) process is used. Specifically, Figure 1 shows an example of a Hewlett-Packard thermal inkjet (TIJ) resistor film stack which is equivalent to that used for TMP resistors [21]. A thin film of tantalum-aluminum (TaAl) is used as the resistive layer which is thermally isolated from the silicon substrate by a thick film of silicon dioxide (SiO${}_{2}$). An aluminum (Al) conductive layer is used to carry the high current needed for rapid heating of the ink to near its critical temperature. An electrical passivation layer of silicon carbide (SiC) and silicon nitride (SiN) is used to isolate the electrical components from the ink. Lastly, a cavitation plate of tantalum (Ta) is used to protect the film stack from the high mechanical stresses associated with thermal bubble cavitation. This detailed film stack enables high repeatability and reliability of TIJ resistors needed for commercial inkjet devices with a lifetime in excess of 10${}^{7}$ pulses before failure; however, we note that initial R&D efforts to prototype a concept do not require commercial product lifetimes. In fact, the ability to rapidly prototype ideas and devices is often of paramount importance in initial R&D learning cycles. To date, nearly all research on high power TIJ/TMP resistors use similar micro-fabrication workflows with inherently long turn around times. Additionally, building complex microfluidic devices such as a microfluidic pneumatic cage [31] often requires lengthy preparation and manufacturing steps involving photoresist or PDMS molds. As such, the present study simplifies the film stack shown in Figure 1 and presents a rapid, low-cost manufacturing process using commercially available substrates and tooling to accelerate thermal bubble-driven micro-pump R&D learning cycles from a matter of weeks/months to hours/days. We anticipate that this rapid fabrication approach will increase accessibility of this technology to researchers as well as speed up initial R&D efforts to assimilate this technology in lab-on-a-chip devices.

## 2. Materials and Methods

### 2.1. Resistor Fabrication

Laser cutting thin films offers a simple, fast non-fab (by which we mean a non semiconductor micro-fabrication workflow, previously successful with sensors, actuators and entire robotic devices [32,33]) approach to fabricate TMP resistors. However, such an approach is inherently single material and care must be taken to ensure electrically isolated cuts with edges as smooth as possible to minimize hot spot locations during heating, as described in Section 2.1.1. Here, we present the use of two commercial laser cutters to fabricate TMP resistors: (1) a *Trotec Speedy 360* CO${}_{2}$/fiber laser and (2) a *Light Conversion CARBIDE-CB5* femtosecond laser system with UV harmonics.

#### 2.1.1. Single Material Resistor Design Optimization

**Figure 2:**
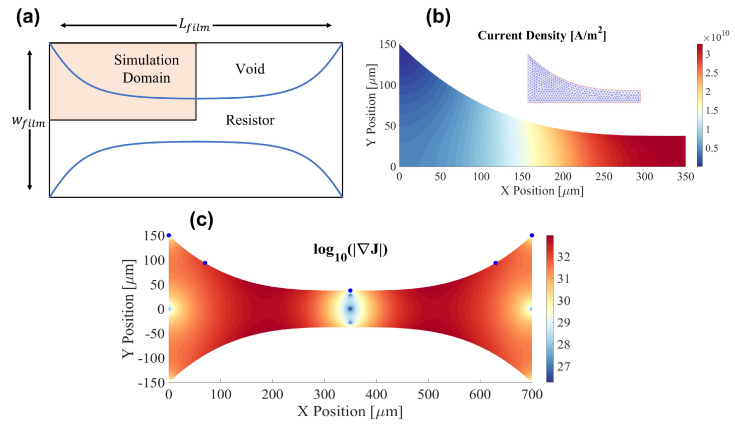
Spline-Based Resistor Topology Optimization—illustrates the resistor design optimization process to minimize thermal stresses using the placement of spline knot points. (**a**) design domain showing exploitation of symmetry in simulation region, (**b**) computational mesh and finite element solution for the current density magnitude ||J||, and (**c**) logscale contour plot of ||∇J|| used to define resistor fitness on optimized designs (sample spline knot points shown).

When designing single material TMP resistors, there exists a large design space to achieve a target end-to-end resistance. We simplify the design space by reducing the resistor definition to a series of splines. In general, not all designs are thermally and mechanically optimal; thermal stresses arising from sharp temperature gradients are the main cause of resistor failure during normal operation [25]. In addition, non-uniform heating will result in localized bubble nucleation in advance of the bulk resistor region which decreases pumping efficiency. As such, resistors should be designed to minimize the temperature gradient across the resistor surface. In this work, we employ a derivative-free design optimization method to discover resistor designs which achieve a target resistance while minimizing sharp temperature gradients. The design space is further restricted to resistors which consist of a solid region and void region separated by a cubic spline and exhibit reflective symmetry in two directions (Figure 2a).

The full dynamics of resistive Joule heating is modeled using a one-way coupling between steady-state direct current conduction (Equation (1)) and unsteady thermal diffusion (Equation (2))

(1) $$\begin{array}{c} \nabla^{2}V=0 \end{array}$$

(2) $$\begin{array}{c} \rho c_{p}\frac{\partial T}{\partial t}=k\nabla^{2}T+Q_{J} \end{array}$$

(3) $$\begin{array}{c} J=-\sigma \nabla V \end{array}$$

(4) $$\begin{array}{c} Q_{J}=\frac{{\left||J|\right|}^{2}}{\sigma } \end{array}$$

where *V* is the electric potential, ρ is the density, $c_{p}$ is the heat capacity, $Q_{f}$ is the volumetric heat generation source due to Joule heating, *k* is the thermal conductivity, *T* is the temperature, σ is the electrical conductivity, and J is the current density. The physics are coupled through the Joule heating source $Q_{J}$. Because the resistor’s in-plane length scale is 3 orders of magnitude larger than in the thickness direction, we consider the resistor to have uniform temperature in the thickness direction and model the problem in 2D. At the length scales (100 μm’s) and power densities (2 × 10${}^{6}$ W/mm${}^{3}$) of TMP resistors in this study, the rate of heat generation by Joule heating dominates the rate of in-plane thermal diffusion by approximately 7 orders of magnitude. As such, during the brief (5 μs) heating pulses, the time-dependent temperature field over the resistor can be simplified to be a linear function of the current density and time. This proportionality means that the temperature gradient of the resistor can be inferred from the gradient of the current density. In this analysis, we take advantage of this correspondence and simplify the model to a 2D electrostatic problem to solve for the current density. Thus, for the optimization process presented here, we seek to minimize the maximum local value of the $L^{2}$ norm of the current density gradient across the surface of the resistor. Optimal designs that minimize this norm exhibit gradual changes in the current density (Figure 2c), while poor designs feature regions of high ||∇J||, corresponding to current crowding and sharp changes in temperature, which create damaging stress in the resistor.

**Figure 3:**
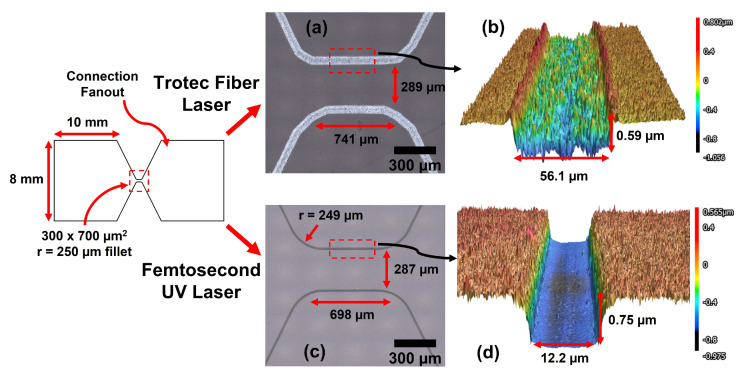
Resistor Fabrication Method and Cut Quality—depicts the laser cutting process and cut quality from both the Trotec fiber laser and femtosecond UV laser systems in a single line vector cut. CAD software, Autodesk Fusion 360, was used to generate a dxf file defining the resistor. (**a**,**b**) the FTO film was cut using the Trotec fiber laser with the following cut settings: power = 20%, speed = 0.71 mm/s (0.02% of max speed), PPI/Hz = 30,000, dpi = 500, and passes = 2. (**a**) illustrates the cut quality from the Trotec fiber laser system. The positioning system lacks sufficient resolution to fully resolve the 250 μm fillet on the resistor edges, but the dimensions of the resistor closely matched that of the dxf design. (**b**) shows the 3D cut profile in which the cut width was 56.1 μm with a depth of 0.59 μm. (**c**,**d**) the FTO film was cut using the femtosecond UV laser with the following cut settings: power = 0.643 W (100%), repetition frequency = 250 kHz, fluence = 7.00 J/cm${}^{2}$, speed = 500 mm/s, and passes = 5. (**c**) shows the cut quality from the femtosecond UV laser system. (**d**) illustrates the 3D cut profile in which the cut width was 12.2 μm with a depth of 0.75 μm. FTO coated glass with a sheet resistance of 8 Ω/sq and a film thickness of 340 nm was used.

The MATLAB constrained nonlinear programming solver *fmincon()* was used to search for high-performing designs which minimize the gradient in current density, allowing the algorithm to specify the positions of knot points that control the cubic spline defining a candidate resistor’s boundary. Equation (1) was solved using the MATLAB Partial Differentiation Toolbox with supply voltage (V = 90 V) and ground (V = 0) boundary conditions applied to the left and right edges of the design boundary respectively (Figure 2b). Our optimization experiments consistently produced designs with electrical resistance equal to the target value ±0.1% and consistently maximized the radius of curvature between the edge of the design domain and the center line. This trend was found across multiple experiments with randomized initial guesses, inclusion/exclusion of tangency constraints at either end of the design domain, and varying numbers of spline control points. As such, to minimize the temperature gradient due to current crowding, single material TMP resistors should be designed such that the transition region from connecting pads to the narrow heater section has as large a radius of curvature as possible. In this study, we use 250 μm radius curves in the transition region (Figure 3).

#### 2.1.2. Laser Cutting of Resistors

TMP resistors were fabricated via laser cutting commercial ITO/FTO thin films sputter coated on glass (Sigma Aldridge, St. Louis, MO, USA). Specifically, a Trotec Speedy 360 and a femtosecond UV laser cutter were characterized and used to create a single line vector cut. The Trotec Speedy 360 laser cutter has a dual source 120 W CO${}_{2}$/50 W fiber laser enabling both cutting/engraving of plastics as well as metal films. The CO${}_{2}$ laser has a wavelength of 10.6 μm and the fiber laser has a wavelength of 1.064 μm. Based on the transmission spectrum of soda lime glass and ITO/FTO films [34,35] as well as the narrower focal diameter of the fiber laser, the fiber laser was used to cut ITO/FTO thin films. Laser cutting settings were determined such that (1) resistors were electrically isolated and (2) cuts were as smooth as possible with minimal damage to the glass substrate. If too much energy is delivered to the glass substrate, it was found that micro-fracturing occurs which creates undesirable hot spot locations during Joule heating leading to resistor failure. As such, we suggest the following vector cut settings for the fiber Trotec system which satisfied both of the aforementioned constraints: power = 20%, speed = 0.71 mm/s (0.02% of max speed, 3.55 m/s), PPI/Hz = 30,000, dpi = 500, and passes = 2. Autodesk Fusion 360 was used to design vector cut files. Figure 3a,b illustrates the cut quality from the vector cut using a Keyence VK X-1100 profilometer. In (a), it was observed that the Trotec positioning system lacks sufficient resolution to fully resolve the curved 250 μm fillet as defined in the CAD design. Additionally, the second pass can occasionally be offset from the first pass creating errors with respect to the design file. However, the positioning system does accurately reflect the desired resistor dimensions of 300 × 700 μm${}^{2}$. In (b), the cut width and depth were measured to be 56.1 μm, implying a minimum feature size of approximately 50 μm, and 0.59 μm respectively. The FTO coated glass had a sheet resistance of 8 Ω/sq with a thickness of 340 nm.

The Light Conversion CARBIDE-CB5 Femtosecond UV laser cutter (herein referred to as the “femtosecond UV laser cutter”) has an ALIO 6-D Hybrid Hexapod stage and integrated SCANLAB excelliSCAN galvanometer positioning system with a spot size of approximately 5 μm enabling precision micro-fabrication of structures on the order of 10’s μm with a galvo repeatability of ±0.4 μrad or ±0.16 μm, at a focal distance of 40 cm. Figure 3c,d illustrates the cut quality from the vector cut using the following laser cutting settings: power = 0.643 W (100%), repetition frequency = 250 kHz, fluence = 7.00 J/cm${}^{2}$, speed = 500 mm/s, and passes = 5. As shown in (c), the fabricated resistor dimensions closely matched that of the CAD design. In (d), the cut width and depth were measured to be 12.2 μm, implying a minimum feature size of approximately 10 μm, and 0.75 μm respectively. Femtosecond UV laser ablation provides a means to rapidly and accurately cut thin films for TMP resistors with near-FAB accuracy due to the short pulse duration (247 fs) of the laser resulting in cold ablation vs. the melt zone formed from longer pulses (67 ns) of the Trotec fiber laser system which causes more damage to the substrate. We note that less expensive UV galvo laser marking tools, such as Full Spectrum Laser MUSE UV Gavlo laser marking system, could potentially be used as a substitute for a femtosecond laser system. The FTO coated glass had a sheet resistance of 8 Ω/sq with a thickness of 340 nm.

### 2.2. Microfluidic Fabrication

**Figure 4:**
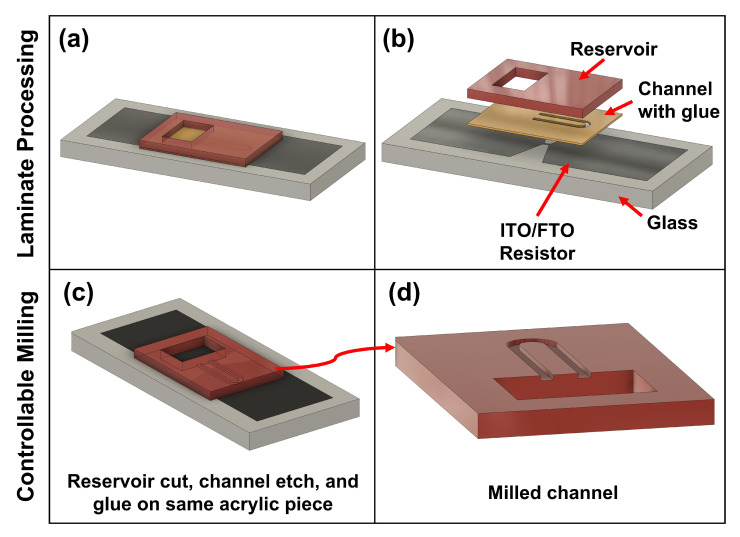
Microfluidic Fabrication Processes—illustrates both laminate and controllable milling microfluidic fabrication processes. (**a**,**b**) show laminate processing in which a 58.42 μm glue layer is laminated to both sides of a 200 μm thick acrylic sheet, and the channel and reservoir layers are defined through laser cutting. (**c**) shows controllable milling on the femtosecond laser cutter system to produce 2.5D microfluidic geometries. Unlike laminate processing, a single acrylic substrate is used upon which the channel and reservoir is defined. (**d**) shows a close up of the milled channel region.

**Figure 5:**
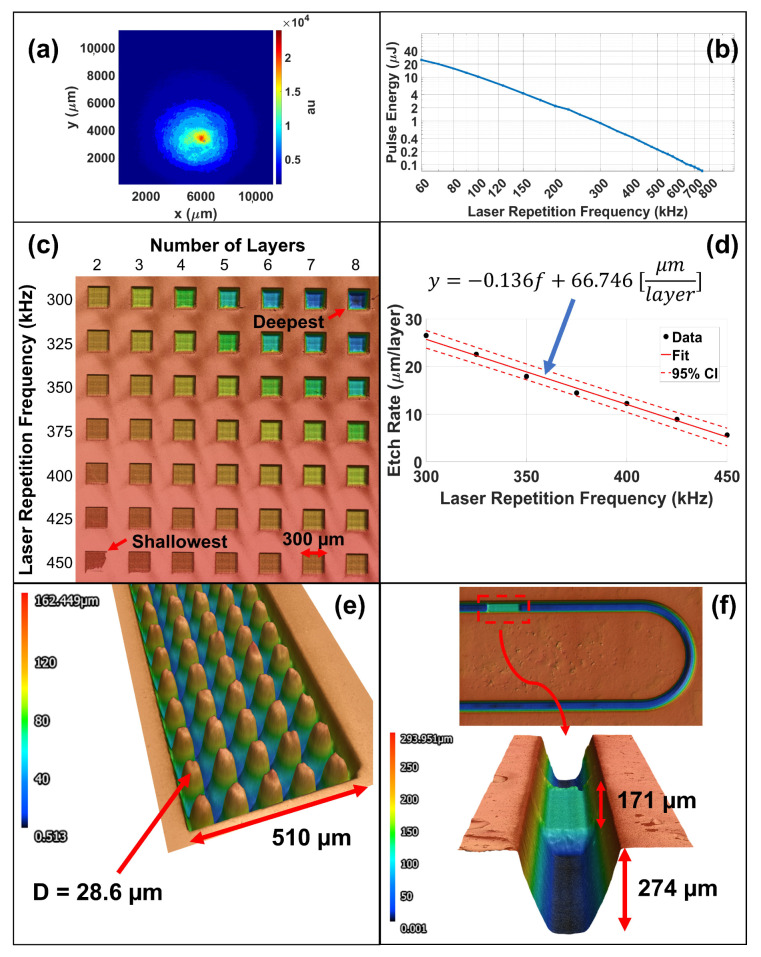
Femtosecond UV Laser Characterization—illustrates laser and etch rate characterization. (**a**) shows the measured beam profile at the lens (which is equivalent to at laser focal plane *without* the 125 mm galvo focusing lens). The beam diameter at the focal plane was calculated to be 8.83 μm from Gaussian beam theory. (**b**) shows the pulse energy as a function of laser repetition frequency. (**c**) shows the etch rate characterization test samples in which 300 × 300 μm${}^{2}$ squares were etched using a cross-hatch line spacing of 3 μm. (**d**) describes the etch rate as a function of laser repetition frequency enabling controllable milling of micro-channels. (**e**,**f**) highlights the ability to fabricate 2.5D micro-structures through controllable milling. (**e**) shows a repeating tapered micro-pillar array of diameter D = 28.6 μm inside a 510 × 126 μm${}^{2}$ channel. (**f**) shows an integrated step height 171 μm from the top channel surface inside a 306 × 274 μm${}^{2}$ channel demonstrating 2.5D milling capability.

Rapid fabrication of microfluidic devices was performed using laminate processing [36] as well as controlled-depth milling of acrylic [37]. Figure 4 illustrates both processes. Laminate processing was performed by laminating a 58.42 μm glue layer (3M 467MP) to both sides of a 200 μm thick acrylic sheet (Emco Industrial Plastics). The laminate sheet was then laser cut to define the microfluidic channel. To form the fluidic reservoir, a 2 mm thick acrylic sheet was laser cut as shown in (a,b). Once cut, the channel layer was manually aligned and applied to the ITO/FTO glass surface upon which the reservoir layer was applied. Laminate processing is a 2D microfluidic fabrication approach in which the channel height is set by the acrylic and glue layer thicknesses. In contrast, controllable milling is a 2.5D microfluidic fabrication process enabling arbitrary channel heights and geometries. In the controllable milling process, a glue layer was laminated to a 2 mm acrylic sheet and a femtosecond UV laser system was used to etch microchannels/micro-structures into the acrylic as well as cut the reservoir shown in (c,d). The milled acrylic was then manually aligned and applied to the ITO/FTO glass surface to define the micro-channel. The femtosecond UV laser cutter enables rapid fabrication of 2.5D micro-channels and micro-structures giving near-FAB precision of microfluidic devices, see Figure 5e,f.

### 2.3. Femtosecond UV Laser Beam Profile and Material Etch Rates

Material cut and etch rates are a function of the laser fluence, see Equation (5).

(5) $$H_{e}=\int_{0}^{\tau }E_{e}\left(t\right)dt=\frac{P\tau }{\pi r^{2}}$$

The fluence is the radiant energy received by a surface per unit area where $H_{e}$ is the fluence [J/m${}^{2}$], $E_{e}$ is the irradiance [W/m${}^{2}$], τ is the pulse duration [s], *P* is the power [W/m${}^{2}$], and *r* is the effective beam radius [m]. Thus, to calculate the fluence of our material cuts and etches, the beam profile and power were first characterized. The beam profile of the femtosecond UV laser cutter was determined using a WinCamD-LCM CMOS beam profiler. Figure 5a shows the resulting beam profile at the lens (which is equivalent the beam profile at the laser focal plane *without* the 125 mm galvo focusing lens). The resulting beam profile has a Gaussian power distribution upon which the effective 1/$e^{2}$ beam diameter was measured to be 6184.6 μm. The Gaussian beam diameter at the focal plane (meaning *with* the 125 mm galvo focusing lens) was calculated to be 8.83 μm using Gaussian beam theory (see Equation (6)) where 2$\omega_{o}$ is the beam diameter at the focal plane, *M* is the beam quality parameter (set as 1), λ is the wavelength (343 nm), *f* is the focal length of the lens, and *D* is the 1/$e^{2}$ beam diameter at the lens [38].

(6) $$2\omega_{o}=\frac{4M^{2}\lambda f}{\pi D}$$

For the beam power, a Newport power meter (model 843-R) was used to measure the power at different laser repetition frequencies at the cutting surface. The femtosecond laser system used a pulse width of 247 fs. Figure 5b shows that the laser power can be approximated as a Sigmoid function in that it logarithmically decays with the laser repetition frequency. Material etch rates were determined by performing a cross-hatch etch with a line spacing of 3 μm at various laser repetition frequencies and number of layers (or passes) as shown in (c). The etch rate was found to be linearly related to the repetition frequency (d).

### 2.4. Electrical Setup

Commonly, TMP resistors (15 × 22.5 μm${}^{2}$) utilize a firing pulse of around 1.5 μs at 30 V to create the heat flux needed to superheat an interfacial layer of fluid above the resistor’s surface for bubble nucleation [5]. For a given material and applied current, as the resistor size increases, the current density decreases and therefore a higher power is needed to reach the critical heat flux for bubble nucleation. In this study, resistor size is on the order of 300 × 700 μm${}^{2}$ with a resistance of 30–50 Ω requiring a firing pulse of 5 μs at 80–100 V for bubble nucleation. Characteristic power and energy demands for this system are: power ≈ 222 W and energy ≈ 1.1 mJ per pulse which corresponds to a heat flux of approximately 1058 W/mm${}^{2}$. In this study, a Sorensen XG300-5.6 power supply was used. Care must be taken with the printed circuit board (PCB) design to enable high voltage (30–300 V), high current (1–4 A), and fast transient (<5 μs) heating pulses. Here, we present a low cost, high power PCB design to implement TMP heating pulses for independent control of 4 TMP resistors. A complete bill of materials and PCB designs are provided in the Matter Assembly Computation Lab’s (https://www.matterassembly.org) (accessed on 25 August 2022) public github repository (https://github.com/MacCurdyLab) (accessed on 25 August 2022) under open access license https://choosealicense.com/licenses/mit/ (accessed on 25 August 2022) as well as the Supplementary Materials.

Figure S5 shows the PCB layout and circuit schematic used to control TMP resistors. KiCAD was used to design the PCB, and an Analog Discovery 2 USB instrument was used as a digital pattern generator. The Analog Discovery 2 has an internal clock of 100 MHz, is programmable, and has 16 digital 3.3 V I/O channels each with a 16 kB buffer making it an ideal low-cost control system. As shown in Figure S5a, the PCB consists of 5 main circuit elements: (1) the digital 3.3 V Analog Discovery input signal is boosted to 15 V using a gate driver for compatibility with the power MOSFET, (2) a low-side current sensor op-amp with a gain of 10.08 is used to measure the resistor’s surface temperature during a heating pulse, (3) power MOSFETs are used to control driving voltage pulses, (4) flyback Schottky diodes are used to minimize inductive ringing caused by long connecting wires to the thin film resistors, and (5) bypass capacitors with bleeder resistors are used to minimize AC noise on high power turn on/off. Figure S5b describes the unit schematic to control a single TMP resistor which can be repeated for independent control of N resistors, in this study N = 4. We stress that the voltages used in this system are *lethal* if handled improperly. The large bypass capacitors (10–220 μF), while needed to filter AC noise on the power lines, increase the danger of the PCB if mishandled. As such, a 3 kΩ, 20 W bleeder resistor is used in order to ensure a discharge time constant of less than 1 s for the system and 2 parallel 100 kΩ, 1 W bleeder resistors are used as safeguards in case the 3 kΩ resistor fails. An emergency off switch is also wired into the main power line to provide an additional level of safety. Apart from hardware safeguards, the PCB is physically isolated and controlled digitally through a custom software graphical user interface, GUI, (described in Figure S2 and provided in the Supplementary Information) to ensure operators are isolated from the system during use.

### 2.5. Imaging Setup

Thermal bubble-driven micro-pumps vaporize a thin layer of fluid creating a vapor bubble which rapidly expands and collapses in less than 50 μs. To fully resolve bubble dynamics, high speed imaging at approximately 1 Mfps is needed. There are two primary means to perform high speed imaging: conventional and stroboscopic. In conventional high speed imaging, a specialized, expensive camera is used to record a *single* transient event in real time. In stroboscopic high speed imaging, an event is reconstructed by taking *multiple* discrete snapshots of repeating events occurring at different time offsets from the start of the event. For events that are not repeatable, conventional high speed imaging is required; for events that are repeatable, such as in the case of TIJ/TMP resistors [15,25], stroboscopic imaging can be used to obtain high resolution, inexpensive, high speed imaging.

In this study, stroboscopic imaging was implemented using a Basler CMOS camera (a2A1920-160 umBAS), which has a global shutter and a minimum exposure time of 1 μs, a reflected light microscopy system (Axiotron ZS-19), and a high intensity pulsed LED illumination source (Lightspeed Technologies HPLS-36DD18B). Figure S1 illustrates the optical setup. The Lightspeed Technologies HPLS-36DD18B LED can be used in continuous output mode or pulsed mode in which short, high intensity light flashes can be produced from 50 ns–2 μs at a 1% duty cycle. The global shutter of the Basler CMOS camera allows the duration of the light pulse to set the effective shutter speed. As such, bubble dynamics can be imaged at a maximum effective frame rate of 20 Mfps with the developed system.

### 2.6. Particle Tracking and Flow Rate Characterization

**Figure 6:**
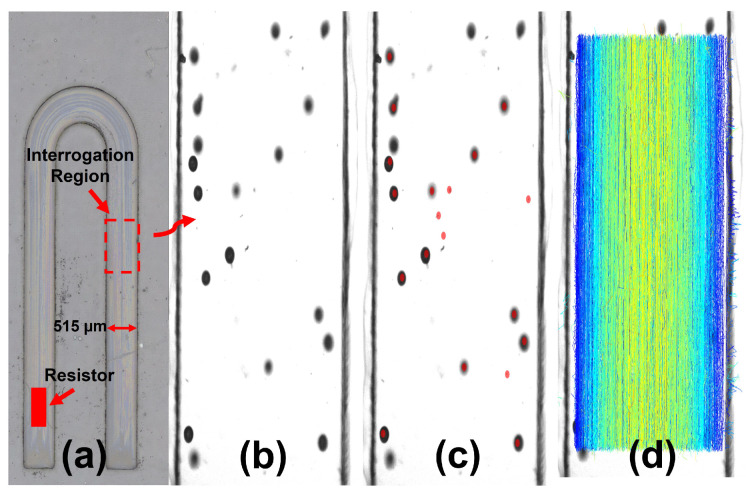
Particle Tracking Process—describes the particle tracking process. (**a**) shows a dry milli-channel of cross-section A = 515 × 315 μm${}^{2}$ and length L = 13.268 mm with a 300 × 700 μm${}^{2}$ TMP resistor placed 1027 μm from the reservoir edge. (**b**) shows neutrally buoyant micro-spheres with a diameter of D = 27–32 μm in a channel filled with water. A MATLAB implementation of the interactive data language, IDL, particle tracking software is used to mark particles (**c**) and link particle movement into trajectories (**d**). Particle trajectories are colored in accordance to average velocity in which the fastest moving particles (shown in yellow with a velocity of 19.2 μm/pulse) are near the center of the channel while the slowest (shown in blue with a velocity of less than 1 μm/pulse) are towards the walls.

Particle tracking is used to estimate the flow rate from thermal bubble-driven micro-pumps in micro/milli-channels. A MATLAB implementation of the interactive data language, IDL, particle tracking software [39] was used to identify and track particle movement. Figure 6 illustrates the particle tracking process. In (a), a thermal bubble-driven micro-pump is placed 1027 μm from the reservoir edge in a micro/mill-channel of cross-section A = width × height = 515 × 315 μm${}^{2}$ and length L = 13.268 mm. In (b), the micro/milli-channel is filled with water and seeded with 27–32 μm diameter neutrally buoyant micro-spheres (Cospheric Inc., Goleta, CA, USA). The particles are identified using image processing (c) and then tracked frame to frame to generate trajectories (d). Particle trajectories are colored in accordance to their average velocities illustrating that the fastest moving particles occurs near the center of the channel which is characteristic of fully developed laminar flow. We note that the developed imaging system measures *per pulse* dynamics and not transient particle displacement dynamics. Therefore, Movie S6 shows particle displacements on a per pulse basis which results in a net forward displacement and does not show the transient N-shaped cumulative flow curve [5] accounting for forward flow during bubble expansion and backwards flow during bubble collapse.

To estimate the flow rate, the average velocity must be determined. The most simplistic approach would be to take the average velocity from all particle trajectories. However, such an approach is only accurate if there exists a uniform sampling of particles in the channel. In practice, this assumption is never valid. Primarily, particles have a finite size and cannot sample the flow near walls. Second, particles can settle and cluster. Third, the particle tracking algorithm ignores particles within one diameter of the image boundary to prevent tracking errors. Also, particles are distributed by the vapor bubble in an unpredictable manner. In our system, the vapor bubble does not take up the entire channel height and such asymmetry can lead to preferential particle distributions; in fact, we observe that there is a higher concentration of particles near the middle of the channel. As such, a more accurate determination of the average velocity, as detailed by Kornilovitch et al. [40], is to measure the maximum particle velocity and use the correspondence between the maximum and average velocity in a rectangular channel, described below in Equations (7)–(9), to estimate the average velocity where *a* is the channel width, *b* is the channel height, $v_{max}$ is the maximum velocity, and $\langle v\rangle$ is the average velocity.

(7) $$\begin{array}{c} v_{max}\left(a,b\right)=\langle v\rangle \cdot \frac{3}{2}\frac{1-\frac{32}{\pi^{3}}\cdot S_{4m}\left(a,b\right)}{1-\frac{192}{\pi^{5}}\frac{b}{a}\cdot S_{3m}\left(a,b\right)} \end{array}$$

(8) $$\begin{array}{c} S_{3m}\left(a,b\right)=\sum_{m=0}^{\infty }\frac{1}{{\left(2m+1\right)}^{5}}tanh\frac{\pi a(2m+1)}{2b} \end{array}$$

(9) $$\begin{array}{c} S_{4m}\left(a,b\right)=\sum_{m=0}^{\infty }\frac{{\left(-1\right)}^{m}}{{\left(2m+1\right)}^{3}cosh\frac{\pi a(2m+1)}{2b}} \end{array}$$

**Figure 7:**
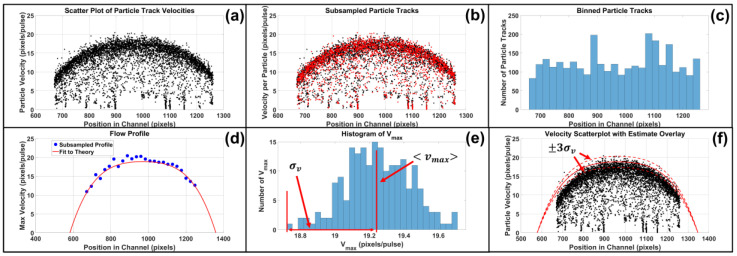
Flow Rate Determination—illustrates the flow rate determination process. (**a**) First, a large data set (2000–3000 tracked particles) of particle location and velocity is generated through the particle tracking process. (**b**) Half of the particle tracks are randomly selected to form a subsample. (**c**) The subsampled particle tracks are grouped into 25 bins of equal width. The number of bins, *k*, is chosen to ensure accurate sampling of the flow profile. (**d**) For each *k^th^* bin, a maximum velocity v${}_{m,k}$ is computed which is taken as an estimate of the flow profile at the bin’s midpoint x${}_{k}$. The set of points {x${}_{k}$,v${}_{m,k}$} is fitted to the theoretical profile of Equation (10) using the channel width (**a**), a horizontal shift (λ), and the overall height ($v_{max}$) as adjustable parameters. The height of the pseudo-parabola fit is the $v_{max}$ of the given subsample. During optimization, we used an asymmetric price function: points below the theoretical curve were priced ten times less than points above the curve since points below the curve could result from insufficient data and artificially depress the sample profile. (**e**) Steps (**b**–**d**) are repeated 200 times producing a distribution of $v_{max}$. The mean value of the distribution is taken as the final estimate of the maximum velocity and the half-width is taken as one standard deviation. (**f**) The original scatter plot data are overlaid with the best-fit theoretical profile with error bounds.

Figure 7 describes this process. (a) First, a large data set, 2000–3000 tracked particles, of particle location and velocity is generated through the aforementioned particle tracking process. (b) Half of the particle tracks are randomly selected to form a subsample. (c) The subsampled particle tracks are grouped into 25 bins of equal width. The number of bins, *k*, is chosen to ensure accurate sampling of the flow profile. (d) For each *k^th^* bin, a maximum velocity v${}_{m,k}$ is computed which is taken as an estimate of the flow profile at the bin’s midpoint x${}_{k}$. The set of points {x${}_{k}$,v${}_{m,k}\}$ is fitted to the theoretical profile of Equation (10) using the channel width (a), a horizontal shift (λ), and the overall height of the pseudo-parabola ($v_{max}$) as adjustable parameters [40].

(10) $$\begin{array}{c} v_{max}\left(x-\lambda \right)=v_{max}\cdot \frac{4\left(\frac{x}{a}\right)\left(1-\frac{x}{a}\right)-\frac{32}{\pi^{3}}\cdot S_{6}\left(x;a,b\right)}{1-\frac{32}{\pi^{3}}\cdot S_{4n}\left(a,b\right)} \end{array}$$

(11) $$\begin{array}{c} S_{4n}\left(a,b\right)=\sum_{n=0}^{\infty }\frac{{\left(-1\right)}^{n}}{{\left(2n+1\right)}^{3}cosh\frac{\pi b(2n+1)}{2a}} \end{array}$$

(12) $$\begin{array}{c} S_{6}\left(x;a,b\right)=\sum_{n=0}^{\infty }\frac{sin\frac{\pi x}{a}\left(2n+1\right)}{{\left(2n+1\right)}^{3}cosh\frac{\pi b(2n+1)}{2a}} \end{array}$$

The height of the pseudo-parabola fit is the $v_{max}$ of the given subsample. During optimization, we used an asymmetric weighting function: points below the theoretical curve were weighted ten times less than points above the curve since points below the curve could result from insufficient data and artificially depress the sample profile. (e) Steps (b–d) are repeated 200 times producing a distribution of $v_{max}$. The mean value of the distribution is taken as the final estimate of the maximum velocity and the half-width is taken as one standard deviation. (f) The original scatter plot data are overlaid with the best-fit theoretical profile with error bounds.

## 3. Results

### 3.1. Electrical Signal Integrity

The Analog Discovery 2 USB instrument can be used as a low-cost, programmable, digital pattern generator. However, it is not intended for direct high power signal control. Fast switching of large currents creates electromagnetic interference (EMI) challenges from both the PCB and MOSFET components. As such, the design of the PCB in terms of component selection and layout is critical to proper signal integrity. We summarize proper board layout and component selection below (also shown in Figure S5) to improve signal integrity:

1. A solid back copper ground plane is used to ensure that the ground is at a common voltage even with large currents flowing.
2. Digital return currents from the Analog Discovery and gate drivers are separated from interfering with the analog return currents from the power MOSFETs. This is done by separate placement of components and traces on the PCB.
3. A 27 Ω series gate resistor is used to “slow down” the power MOSFET turn on/off time to reduce gate ringing.
4. Gate drivers with internal Miller Clamps are used to reduce capacitive ringing on turn off.
5. Flyback Schottky diodes are used to minimize inductive ringing caused by connecting wires from the main power supply to the thin film resistors.
6. Large bypass capacitors (100 μF and 10 μF) are used to maintain a steady supply voltage and provide large, transient current draws thus reducing transient spikes from the power supply.
7. Smaller bypass capacitors (0.1 μF and 10 μF) are used to stabilize the supply voltage to the gate driver and mitigate high frequency voltage spikes in the power supplies.

Ultimately, the PCB and control electronics are used to generate high power, transient voltage pulses to drive TMP resistors. A detailed analysis of signal integrity and the developed PCB board can be found in Figures S5 and S6.

### 3.2. Temperature Sensing

In-situ temperature sensing of a TMP resistor during a driving pulse enables direct electrical measurement of both time of nucleation onset (needed to find suitable firing parameters) as well as the fluid temperature at nucleation (important for estimating the vapor bubble’s initial temperature). Subregion 2 of Figure S5a shows the temperature sense circuit used in this study. A differential op-amp with a gain of 10.08 was used in a low-side current sense setup to amplify the voltage drop across a 104.553 mΩ sense resistor. Knowing the voltage drop across the TMP resistor and the current passing through the system, the resistance of the TMP resistor as a function of time was measured. The resistance can be related to the resistor’s surface temperature assuming a linear temperature coefficient of resistance shown in Equation (13)

(13) $$R\left(T\right)-R_{p}=R_{o}\left[1+\alpha \left(T-T_{o}\right)\right]$$

where *R* is the measured resistance, $R_{p}$ is the parasitic resistance, $R_{o}$ is the initial resistance, α is the temperature coefficient of resistance (TCR), *T* is the resistor surface temperature, and $T_{o}$ is the initial temperature. We note that the resistance measurement is a lump sum of both the connection leads and the heating region. Since only the heating region will change resistance, the parasitic resistance ($R_{p}$) due to the connections is subtracted from the measured resistance.

**Figure 8:**
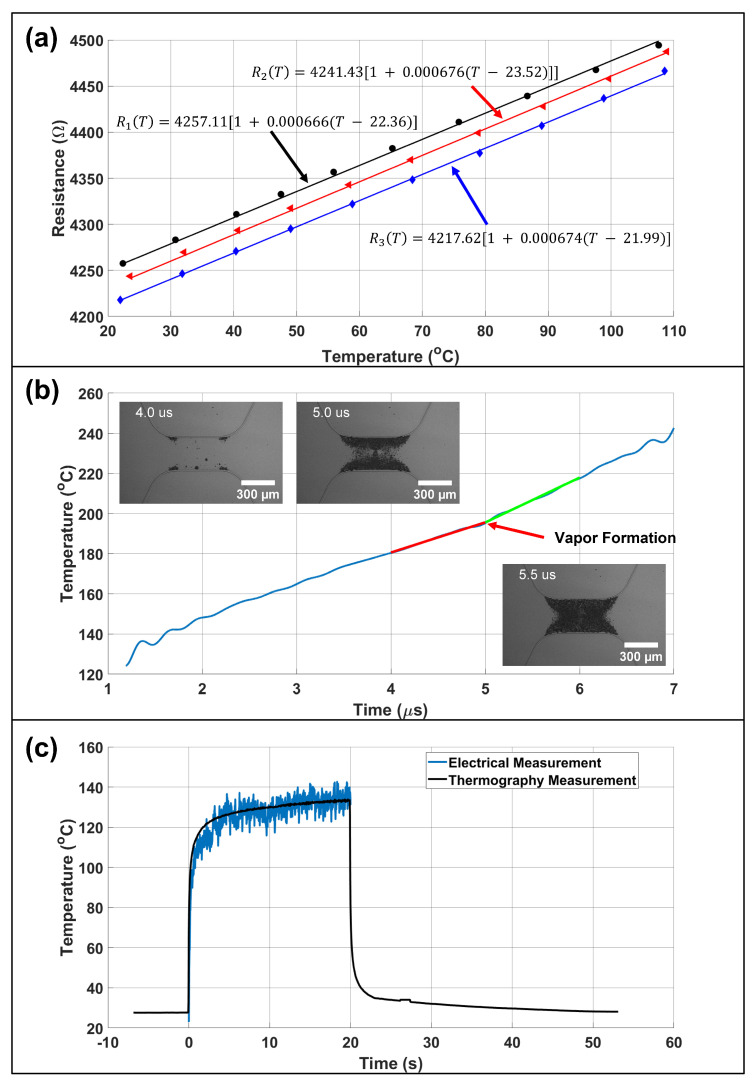
TMP Resistor Surface Temperature—shows the temperature coefficient of resistance (TCR) and surface temperature measurements. (**a**) illustrates TCR measurements for 3 resistors on separate FTO 8 Ω/sq substrates to determine inter-sample uniformity. The TCR value for FTO 8 Ω/sq substrates was 6.72 × 10${}^{-4}$± 5.37 × 10${}^{-6}$ [1/${}^{\circ }$C]. (**b**) depicts the surface temperature of an R = 48.32 Ω, 300 × 700 μm${}^{2}$ FTO TMP resistor during a 8 μs, 100 V heating pulse. A low pass Butterworth filter of order 12 with a half power frequency of 3.33 MHz was used to remove signal noise. Oscilloscope data was recorded at 2 GHz, with N = 64 sample averaging. Insets show stroboscopic images at t = 4 μs, the onset of bubble nucleation, t = 5 μs, and t = 5.5 μs, full vapor layer formation. Once the vapor layer forms, the heat transfer coefficient is significantly reduced resulting in a change of slope at approximately t = 5.0 μs denoted by the highlighted tangent lines. (**c**) shows agreement between electrical and thermography measurements for the resistor surface temperature when fired in air with a 20 s, 6.5 V heating pulse. Electrical measurement data was filtered with a moving average.

The TCR value was found by placing FTO resistors in a temperature controllable furnace from 23–110 ${}^{\circ }$C with 10 ${}^{\circ }$C ramp steps. Figure 8a shows the TCR values for 3 resistors on separate FTO 8 Ω/sq substrates to test for inter-sample TCR uniformity. The TCR value for FTO 8 Ω/sq substrates was 6.72 × 10${}^{-4}$± 5.37 × 10${}^{-6}$ [1/${}^{\circ }$C]. Once known, the TCR value was used to estimate an R = 48.32 Ω, 300 × 700 μm${}^{2}$ FTO TMP resistor’s surface temperature during a 8 μs, 100 V heating pulse in water as shown in Figure 8b. A low pass Butterworth filter of order 12 with a half power frequency of 3.33 MHz was used to remove signal noise. Oscilloscope data was recorded at 2 GHz, with N = 64 sample averaging. The vapor layer begins to form at t = 4.0 μs and fully forms over the resistor’s surface at t = 5.0 μs. Once the vapor layer fully forms, the heat transfer coefficient is significantly reduced resulting in a change of slope at t = 5.0 μs denoted by the tangent lines. The resistor’s surface temperature at full vapor layer formation was 195 ${}^{\circ }$C which is comparable to that in literature for TMP resistors [41]. To verify the accuracy of electrical temperature sensing, a FLIR T650sc thermal camera was used to measure the surface temperature of a R = 45.50 Ω, 300 × 700 μm${}^{2}$ FTO TMP resistor during a 20 s, 6.5 V heating pulse in air. The longer pulse duration at a lower voltage was used to avoid resistor burnout, defined as the point at which the resistor fails, while acquiring sufficient thermography data to compare to electrical data since the FLIR T650sc thermal camera has a maximum frame rate of 60 Hz. As shown in Figure 8c, electrical temperature sensing is in agreement with thermography data where the maximum resistor surface temperature over time was analyzed. Electrical measurement data was filtered with a moving average of 50 samples.

### 3.3. Open Reservoir vs. Confined Bubble Dynamics

**Figure 9:**
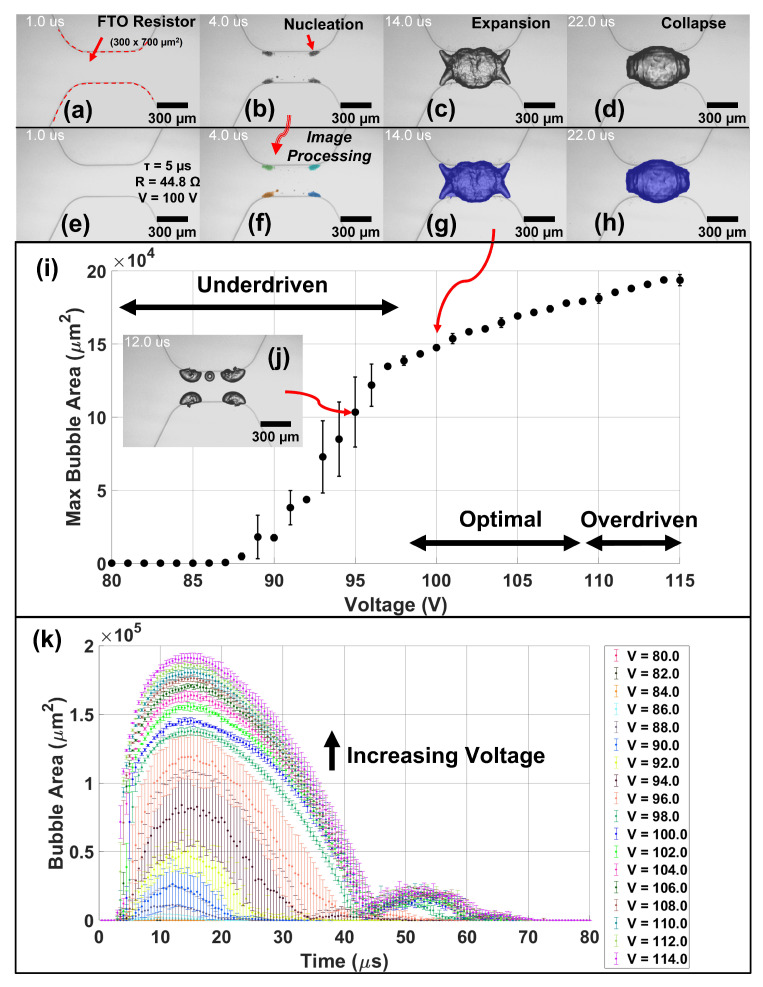
Open Reservoir Femtosecond Resistor Characterization *Inter-Device Reproducibility*—illustrates the effect of voltage on the maximum bubble area for a 300 × 700 μm${}^{2}$ FTO (8 Ω/sq) resistor in water with a 250 μm fillet and firing parameters as follows: pulse duration (τ) = 5 μs, firing frequency (f) = 10 Hz, and resistance (R) = 44.8 Ω. (**a**–**d**) Show the bubble evolution over time in which (**e**–**h**) show the calculation of bubble area using background subtraction image processing. (**i**) Depicts the maximum bubble area as a function of applied voltage with inset (**j**) showing the maximum bubble area at t = 12 μs for V = 95 V. (**k**) Illustrates the full time history of the bubble area during expansion, collapse, and rebound phases as a function of voltage. Stroboscopic imaging was performed using a 1 μs exposure (“shutter”) time with a 500 ns light pulse for an effective 2 Mfps frame rate. N = 3 sample replicates were performed for each voltage level in which each sample is a different resistor.

**Figure 10:**
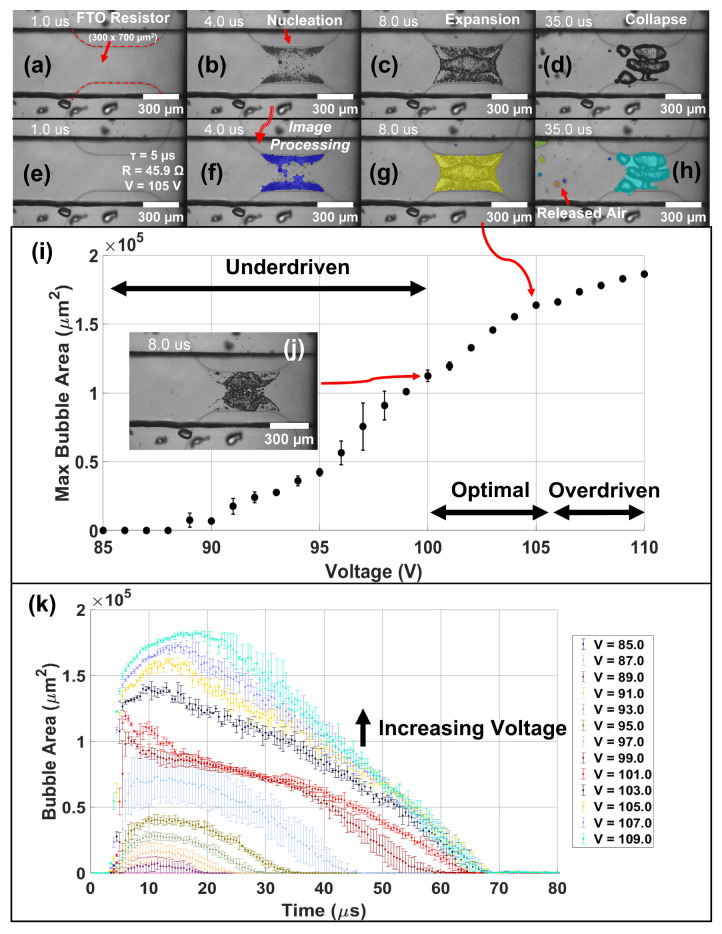
Closed Channel Femtosecond Resistor Characterization *Intra-Device Reproducibility*—illustrates the effect of voltage on the maximum bubble area for a 300 × 700 μm${}^{2}$ FTO (8 Ω/sq) resistor confined in a 515 × 315 μm${}^{2}$ U-shaped channel, as shown in Figure 6a, filled with water. The resistor has a 250 μm fillet and firing parameters were as follows: pulse duration (τ) = 5 μs, firing frequency (f) = 10 Hz, and resistance (R) = 45.9 Ω. (**a**–**d**) Show the bubble evolution over time in which (**e**–**h**) show the calculation of bubble area using background subtraction image processing. (**i**) Depicts the maximum bubble area as a function of applied voltage with inset (**j**) showing the maximum bubble area at t = 8 μs for V = 100 V. (**k**) Illustrates the full time history of the bubble area during expansion and collapse phases as a function of voltage. Stroboscopic imaging was performed using a 1 μs exposure time with a 500 ns light pulse for an effective 2 Mfps frame rate. N = 3 sample replicates were performed for each voltage level in which each sample is a different cycle of the same resistor.

Since the expansion and collapse phase of the vapor bubble drives fluid motion, it is important to study the bubble dynamics of fabricated TMP resistors. Here, we characterize 300 × 700 μm${}^{2}$ FTO TMP resistors in both an open reservoir, when water is simply placed over the resistor, and when confined to a channel, as is the case during micro-pump operation. Femtosecond laser cut resistors are shown in Figure 9 and Figure 10 while Trotec fiber laser cut resistors are shown in Figures S3 and S4 of the Supplementary Information. Femtosecond laser cut resistors were found to be the most robust due to smooth cut edges which minimizes hot spots and failure points. Characterization of TMP resistors was performed using a custom low-cost (<$4500) imaging system detailed in Figure S1 and Table S1 of the Supplementary Information. A full bill of material for the stroboscopic imaging components is included in Table S1 as well as a custom GUI which controls the imaging system. Open reservoir bubble dynamics, shown in Figure 9a–d, depict snapshots of vapor bubble evolution over time for an FTO 8 Ω/sq, R = 44.8 Ω TMP resistor in water driven with a 5 μs firing pulse and imaged with a 500 ns light pulse for an effective 2 Mfps frame rate. Bubble area is computed in (e–h) using a custom MATLAB background subtraction image processing code. The firing voltage is swept from 80 to 115 V in (i) to determine the optimal firing parameters. A TMP resistor is underdriven when the vapor bubble is not fully formed over the surface of the resistor, as shown in inset (j), and is overdriven when excess energy is applied after full vapor bubble formation leading to only slight increases in the maximum bubble area. TMP resistors operating in the overdriven regime can suffer from burnout, occurring around 140 V in these designs, and thus it is desirable to operate TMP resistors in the optimal regime between being underdriven and overdriven. (k) illustrates the full time history of the bubble area during expansion, collapse, and rebound phases as a function of firing voltage. The rebound phase occurs when the vapor bubble collapses and creates sudden regions of local low pressure creating post-collapse cavitation events. In an open reservoir, bubble expansion and collapse takes approximately 40 μs and the rebound phase ends at approximately 60 μs for well developed drive bubbles. Error bars are calculated from N = 3 repeats in which each sample is a different resistor. Thus, there is a high degree of *inter-device* repeatability.

Confined vapor bubbles exhibit different bubble dynamics than those in an open reservoir. Namely, in an open reservoir, the vapor bubble is purely inertially driven; in a closed channel, the vapor bubble is confined and the local pressures developed can cause secondary cavitation events and non-uniformities in the vapor bubble. Figure 10 illustrates the characterization process for a 300 × 700 μm${}^{2}$ FTO TMP resistor in a 515 × 315 μm${}^{2}$ channel filled with water. (a–d) depict snapshots of vapor bubble evolution over time for a R = 45.9 Ω, FTO 8 Ω/sq TMP resistor in water driven with a 5 μs firing pulse and imaged with a 500 ns light pulse for an effective 2 Mfps frame rate. Bubble area is computed in (e–h) using a custom MATLAB background subtraction image processing code. We note that in (c,d), the vapor bubble breaks up and does not stay as a single bubble as in the open reservoir case. In addition, air bubbles are formed from secondary cavitation events shown in (h) which remain suspended in the fluid. The firing voltage is swept from 85 to 110 V in (i) to determine the optimal firing parameters. Inset (j) shows a fully formed vapor bubble at the lower end of the optimal firing regime. (k) illustrates the full time history of the bubble area during expansion and collapse phases as a function of firing voltage. Vapor bubble expansion and collapse takes approximately 70 μs for well developed drive bubbles. Error bars are calculated from N = 3 repeats in which each sample is a different cycle of the same resistor. Thus, there is also a high degree of *intra-device* repeatability in addition to *inter-device* repeatability.

### 3.4. Thermal Bubble-Driven Micro-Pumps

**Figure 11:**
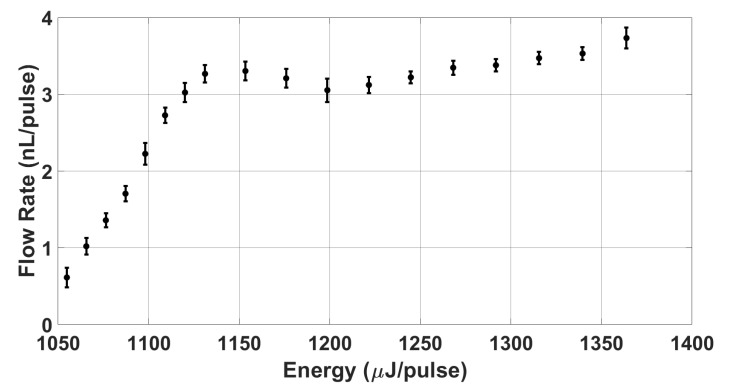
Micro-Pump Flow Rate vs. Energy Analysis—shows the flow rate saturation behavior of thermal bubble-driven micro-pumps. A 300 × 700 μm${}^{2}$ FTO 8 Ω/sq TMP resistor of R = 45.9 Ω is placed 1027 μm from the end of a length L = 13.268 mm channel of cross-sectional area A = 515 × 315 μm${}^{2}$ as shown in Figure 6a. Firing voltage was varied from 98.50 to 112 V with a 5 μs pulse duration corresponding to energies of 1055 to 1364 μJ/pulse. The firing frequency was 20 Hz. At the lower bound, the flow rate becomes 0 when applied energy no longer forms a vapor bubble; at the upper bound, the flow rate slightly increases with applied energy until the resistor burns out and fails.

The previous sections have detailed each individual component (TMP driving electronics, stroboscopic imaging, resistor fidelity, and particle tracking) needed to study and apply TMP resistors as micro-pumps. Here, we demonstrate that laser cut ITO/FTO thin films can serve as a means to rapidly fabricate low-cost thermal bubble-driven micro-pumps. Namely, we characterize the pumping effect of a 300 × 700 μm${}^{2}$ FTO 8 Ω/sq TMP resistor placed 1027 μm from the end of a length L = 13.268 mm channel of cross-sectional area A = 515 × 315 μm${}^{2}$ as shown in Figure 6a. Figure 11 illustrates that the flow rate initially increases as the TMP resistor transitions from being underdriven to being optimally driven when the vapor bubble becomes fully developed (E = 1087 μJ/pulse, V = 100 V, f = 20 Hz). As the resistor is overdriven, the flow rate increases marginally and begins to saturate at approximately 3.34 nL/pulse (66.8 nL/s). The addition of more energy after saturation causes a slightly larger nucleation area on the resistor which accounts for the observed increase in flow rate when overdriven. We note that these flow rates are on the order of 400× larger than that previously demonstrated by Kornilovitch et al. in which flow rates were on the order of 8 pL/pulse [2]. In general, these results indicate that TMP resistors can be used to generate flow rates on the order of pL/pulse to nL/pulse depending on the microfluidic application. Thus, it could one day be advantageous to integrate different sizes of TMP resistors to enable specific flow rates depending on the microfluidic application or channel length of a microfluidic system.

## 4. Conclusions

The present study demonstrates a rapid, low-cost means of fabricating TMP resistors by laser cutting commercial, single material thin films. High power driving electronics and software were developed along with a stroboscopic imaging system to characterize TMP resistors and their operation as thermal bubble-driven micro-pumps. Driving electronics were capable of generating 0–300 V, >200 ns voltage pulses along with simultaneous sensing of a TMP resistor’s surface temperature. It was found that the commercial FTO 8 Ω/sq thin films (Sigma Aldridge, St. Louis, MO, USA) had high TCR uniformity across substrates with an average TCR value of 6.72 × 10${}^{-4}$± 5.37 × 10${}^{-6}$ [1/${}^{\circ }$C] enabling reliable surface temperature measurements. Full vapor formation occurred at a surface temperature of 195 ${}^{\circ }$C during a 8 μs, 100 V heating pulse of a 300 × 700 μm${}^{2}$ FTO resistor. Thermal bubble-driven micro-pump operation of our low-cost, rapid, single film TMP resistors was demonstrated with a saturated flow rate of approximately 3.34 nL/pulse (66.8 nL/s). Single material commercial thin film processing of TMP resistors enabled fabrication of thermal bubble-driven micro-pumps in a matter of hours/days dramatically accelerating learning cycles compared to traditional micro-fabrication approaches.

Thermal bubble-driven micro-pumps hold great promise in enabling very large-scale integration (VLSI) of microfluidic systems for both chemical/biological processing and lab-on-a-chip devices. The micro-pump technology described in this paper is in its infancy, and therefore the ability to rapidly prototype designs is paramount to understanding and applying such pumps to real world applications like lab-on-a-chip devices. In this work, we detail a simple, rapid, low-cost fabrication approach for thermal bubble-driven micro-pumps and associated microfluidics to accelerate thermal bubble-driven micro-pump R&D learning cycles from a matter of weeks/months to hours/days. All driving electronics, software, and imaging approaches in this work are available via the open-source Creative Commons Attribution 3.0 Unported License (https://choosealicense.com/licenses/mit/ (accessed on 25 August 2022)) to make thermal bubble-driven micro-pumps more readily available.

## Data Availability

Data is available at reasonable request to the corresponding author.

## Supplementary Materials

The following are available online at https://www.mdpi.com/article/10.3390/mi13101634/s1. Figure S1: Imaging and controller schematic; Figure S2: Controller GUI; Figure S3: Open channel Trotec resistor characterization; Figure S4: Closed channel Trotec resistor characterization; Figure S5: Electrical driving circuit; Figure S6: Electrical signal integrity; Figure S7: 50 × 100 μm resistor; Table S1: Bill of materials summary; Movie S1: Stroboscopic imaging of a femtosecond laser fabricated resistor in water, no channel; Movie S2: Stroboscopic imaging of a femtosecond laser fabricated resistor in water, with channel; Movie S3: Stroboscopic imaging of a Trotec fiber laser fabricated resistor in water, no channel; Movie S4: Stroboscopic imaging of a Trotec fiber laser fabricated resistor in water, with channel; Movie S5: FLIR thermography imaging of a resistor heating pulse; Movie S6: Particle tracking video example; Bill of Materials S1: Lists the Mouser components used in the PCB; PCB Layout File S1: PCB layout file used for ordering through OSH Park; Controller GUI S1: Folder containing python GUI codes used to control the experimental setup.
