# Supplementary material for: Rapid Fabrication of Low-Cost Thermal Bubble-Driven Micro-Pumps
Source: Micromachines (Basel). 2022 Sep 29;13(10):1634. doi: 10.3390/mi13101634 (PMC9610814; doi:10.3390/mi13101634)
Supplement: Supplementary file 1 [file micromachines-13-01634-s001.zip › Supplemental Information.pdf]

Supplementary Materials for

Rapid Fabrication of Low-Cost Thermal Bubble-Driven Micro-Pumps

Brandon Hayes, Lawrence Smith, Heiko Kabutz, Austin C. Hayes, Gregory L. Whiting, Kaushik Jayaram, and Robert MacCurdy

The PDF file includes:

- fig. S1. Imaging and Controller Schematic
- fig. S2. Controller GUI
- fig. S3. Open Channel Trotec Resistor Characterization
- fig. S4. Closed Channel Trotec Resistor Characterization
- fig. S5. Electrical Driving Circuit
- fig. S6. Electrical Signal Integrity
- fig. S7. 50 x 100 μm Resistor
- table S1. Bill of Materials Summary

Other Supplementary Material for this manuscript includes the following:

- movie S1 (.mp4). Stroboscopic imaging of a femtosecond laser fabricated resistor in water, no channel.
- movie S2 (.mp4). Stroboscopic imaging of a femtosecond laser fabricated resistor in water, with channel.
- movie S3 (.mp4). Stroboscopic imaging of a Trotec fiber laser fabricated resistor in water, no channel.
- movie S4 (.mp4). Stroboscopic imaging of a Trotec fiber laser fabricated resistor in water, with channel.
- movie S5 (.mp4). FLIR thermography imaging of a resistor heating pulse.
- movie S6 (.avi). Particle tracking video example.
- bill of materials S1 (.xls). Lists the Mouser components used in the PCB
- PCB layout file S1 (.kicad_pcb). PCB layout file used for ordering through OSH Park
- controller GUI S1 (.zip). Folder containing python GUI codes used to control the experimental setup

Supplementary Materials

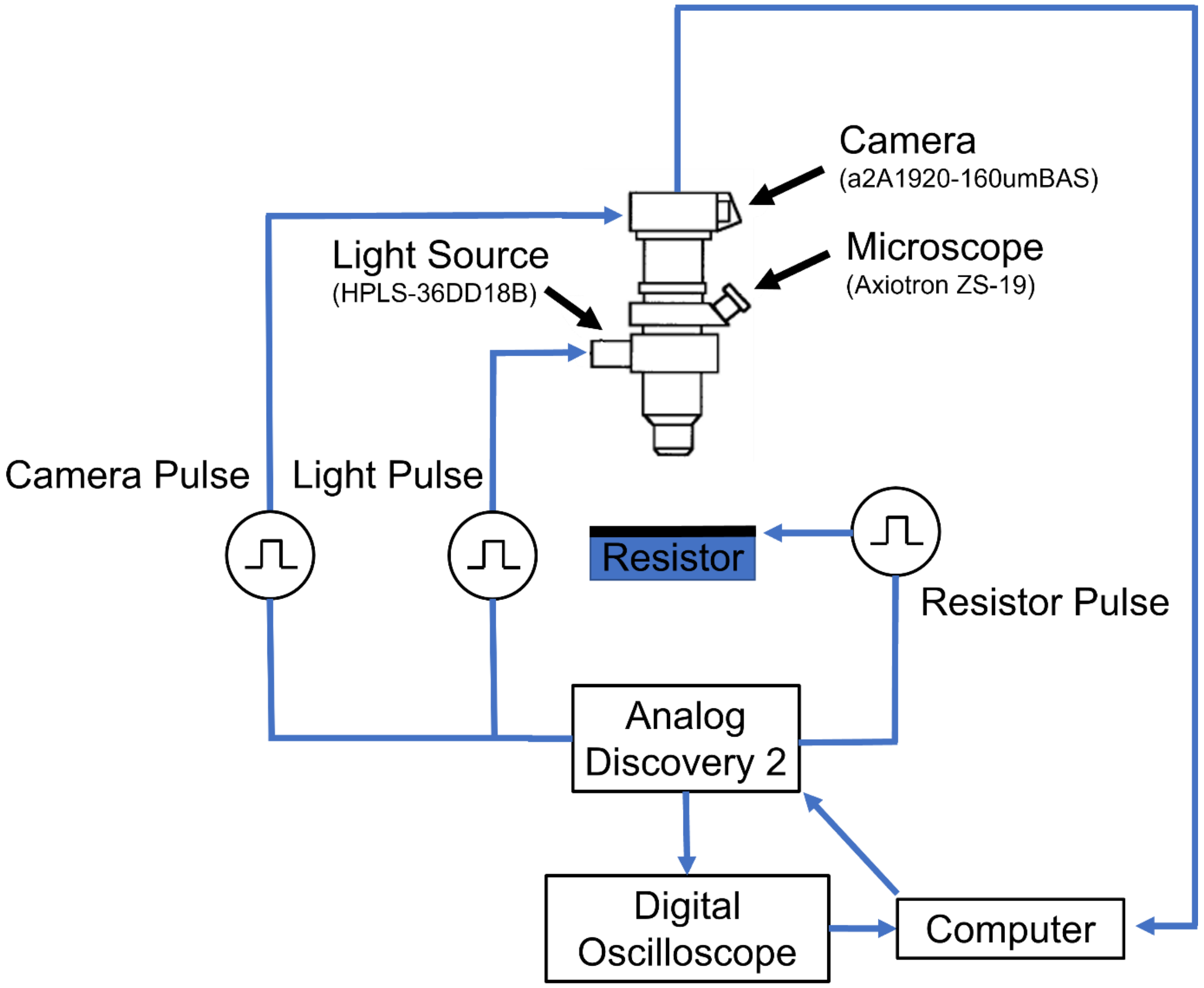

fig. S1. Imaging and Controller Schematic. Stroboscopic imaging is performed using a reflective light microscope (Axiotron ZS-19) system. A mirror can be placed underneath the ITO/FTO coated glass if more light is needed to be captured. An Analog Discovery 2 USB instrument is used to send timing pulses to the camera (Basler a2A1920-160umBAS), light source (HPLS-36DD18B), digital oscilloscope, and resistor (coupled to PCB described in Figure 6). Timing signal synchronization is critical for stroboscopic imaging of thermal bubbles.

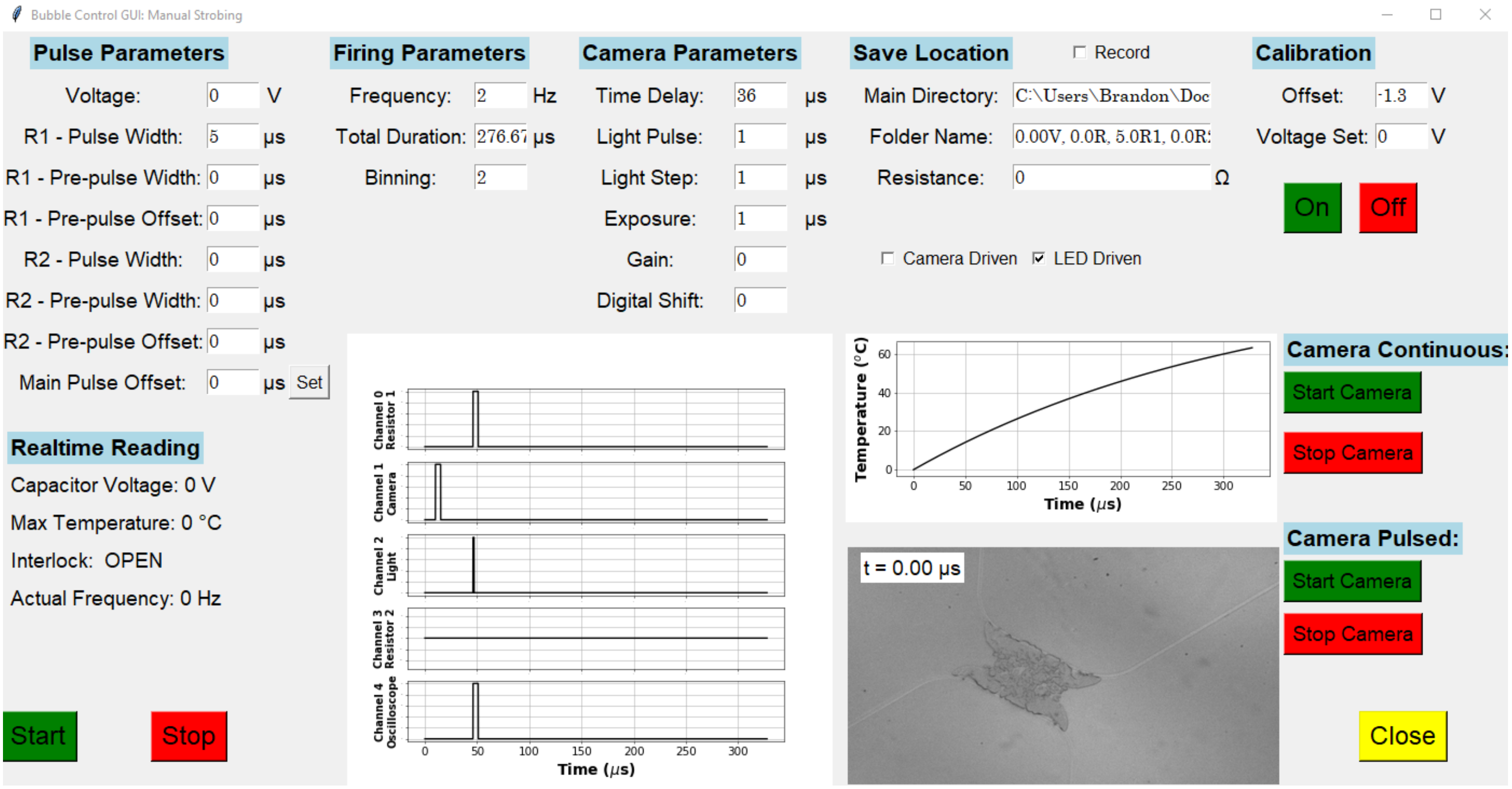

fig. S2. Controller GUI. Sample python GUI for two resistor control stroboscopic imaging. *Pulse Parameters* allow specification of main power supply voltage and pulse parameters in which pre-pulses and delays can be set. *Firing Parameters* allow specification of the firing frequency, total imaging time, and camera binning. *Camera Parameters* allow configuration of the camera delay (intrinsic to the Basler camera), light pulse, and camera exposure, gain, and digital shift. *Save Location* allows selection of the file save location along with the resistance of the TMP resistor and objective magnification used. The system can be set for camera driven stroboscopy, which has a minimum shutter of 1 μs, or HPLS-36DD18B light source driven stroboscopy, which has a minimum pulse width of 50 ns. *Calibration* provides a means to calibrate the power supply voltages if shifts occur over time. *Camera Continuous* toggles displaying the camera output in continuous mode. *Camera Pulsed* toggles displaying the camera output in pulsed mode.

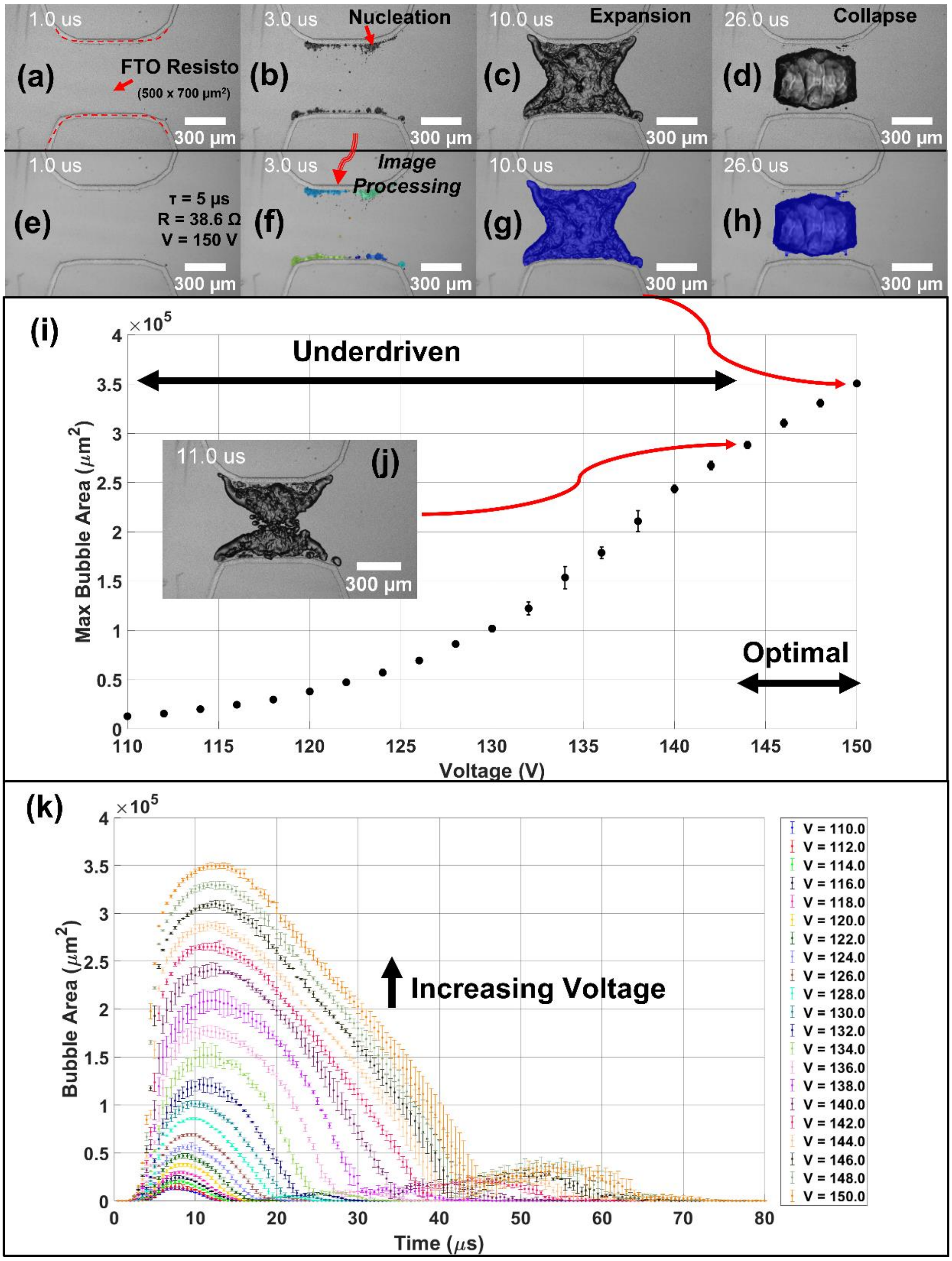

fig. S3. Open Channel Trotec Resistor Characterization. Illustrates the effect of voltage on the maximum bubble area for a 500 x 700 μm^2 FTO (8 Ω/sq) resistor in water with a 250 μm fillet and firing parameters as follows: pulse duration (τ) = 5 μs, firing frequency (f) = 10 Hz, and resistance (R) = 38.6 Ω. (a-d) Show the bubble evolution over time in which (e-h) show the calculation of bubble area using background subtraction image processing. (i) Depicts the maximum bubble area as a function of applied voltage with inset (j) showing the maximum bubble area at t = 11 μs for V = 144 V. (k) illustrates the full time history of the bubble area during expansion and collapse phases as a function of voltage. Stroboscopic imaging was performed using a 1 μs exposure time with a 500 ns light pulse for an effective 2 Mfps frame rate.

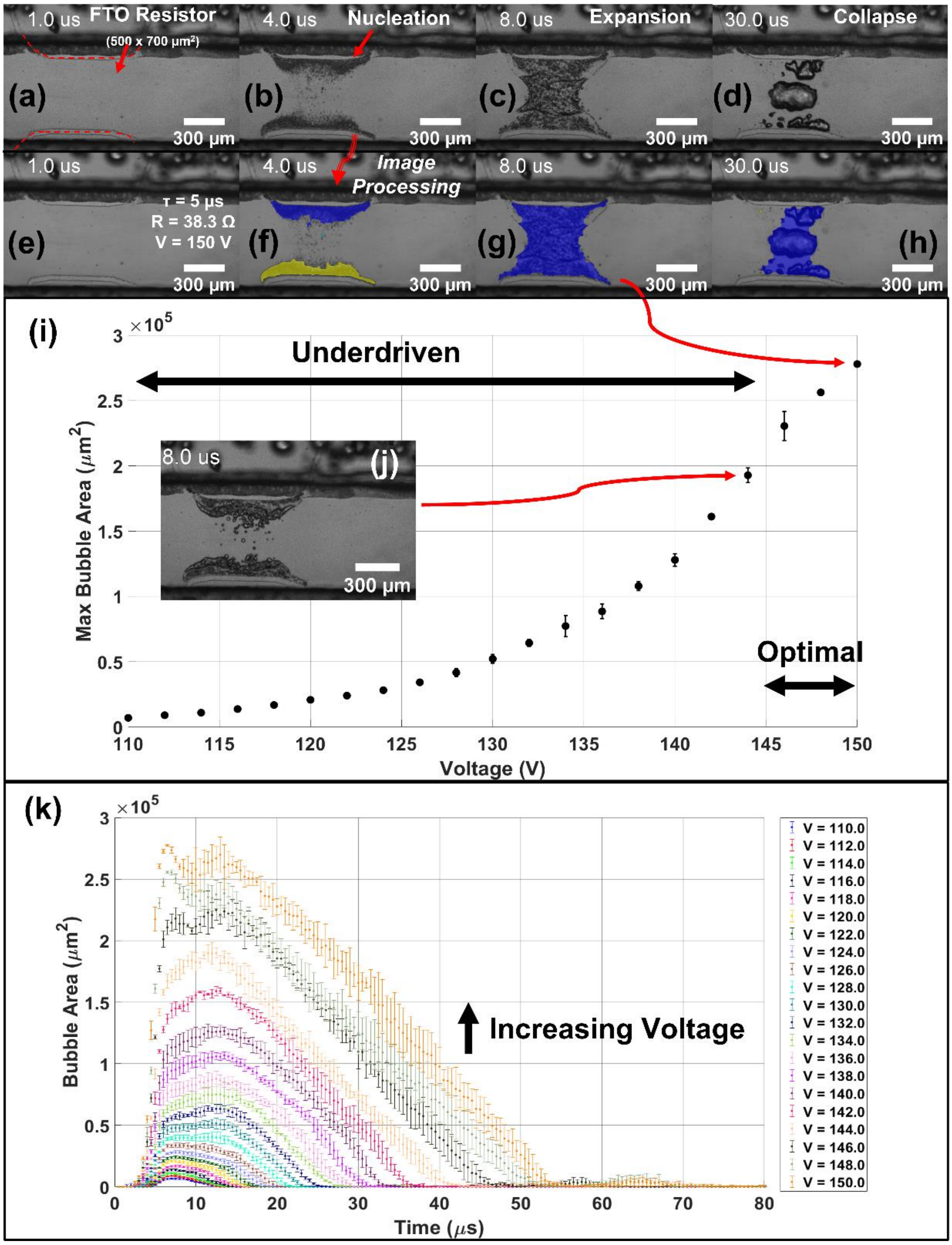

fig. S4. Closed Channel Trotec Resistor Characterization. Illustrates the effect of voltage on the maximum bubble area for a 500 x 700 μm^2 FTO (8 Ω/sq) resistor in water confined to a 340 x 730 μm^2 U-shaped channel with a 250 μm fillet and firing parameters as follows: pulse duration (τ) = 5 μs, firing frequency (f) = 10 Hz, and resistance (R) = 38.3 Ω. (a-d) Show the bubble evolution over time in which (e-h) show the calculation of bubble area using background subtraction image processing. (i) Depicts the maximum bubble area as a function of applied voltage with inset (j) showing the maximum bubble area at t = 8 μs for V = 144 V. (k) Illustrates the full time history of the bubble area during expansion and collapse phases as a function of voltage. Stroboscopic imaging was performed using a 1 μs exposure time with a 500 ns light pulse for an effective 2 Mfps frame rate.

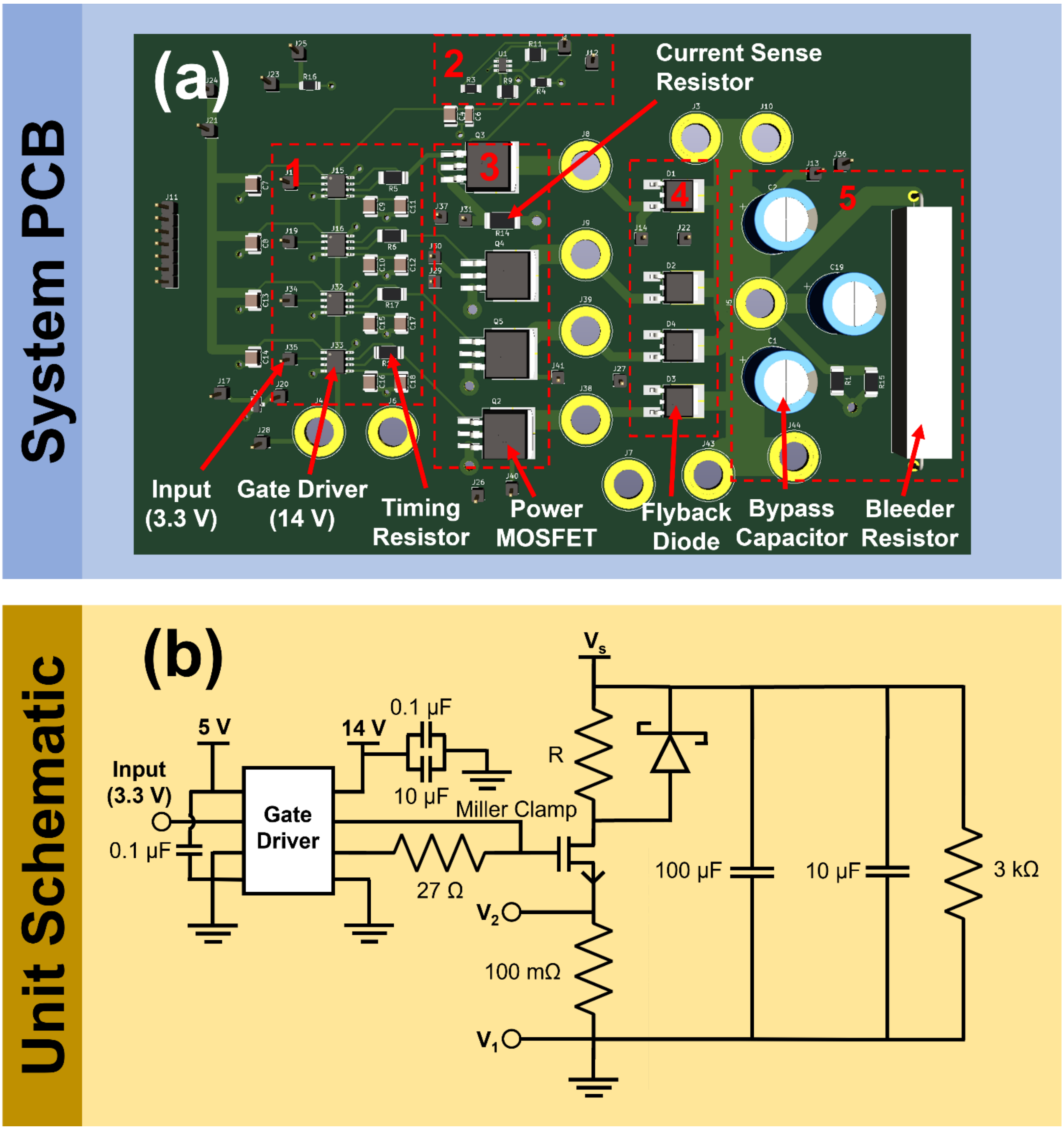

fig. S5. Electrical Driving Circuit. Illustrates the PCB layout and unit resistor controller schematic to drive transient, high power TMP electrical pulses. (a) shows the system PCB layout to independently control 4 TMP resistors. Subregions 1-5 highlight key circuit elements: (1) the digital 3.3 V Analog Discovery input signal is boosted to 15 V using a gate driver for compatibility with the power MOSFET, (2) a low-side current sensor op-amp (connected between V_1 and V_2) is used to measure the resistor's surface temperature during a heating pulse, (3) power MOSFETs are used to control driving voltage pulses, (4) flyback Schottky diodes are used to minimize inductive ringing caused by long connecting wires to the thin film resistors, and (5) bypass capacitors with bleeder resistors are used to minimize AC noise on high power turn on/off. (b) shows the unit schematic used to control a single heating resistor (R) which can be repeated for control of N resistors; in this study N = 4.

Here, we demonstrate the signal integrity for a high power voltage pulse of V = 105 V and current I = 2.15 A was passed through a 300 µm x 700 µm ITO TMP resistor with a water film over its surface. As shown in figure S6 large, transient currents induce electromagnetic interference on the digital 3.3 V Analog Discovery input signal (a-b); however, such noise is nearly eliminated on the output gate driver signal that is sent to the power MOSFET gate. The rise and fall time for the system were 48 ns and 93 ns respectively to reach the power MOSFET 5 V threshold voltage to fully turn on. As such, the developed PCB shown in figure S5 serves as a reference design for users who need to generate similar transient voltage pulses to drive TMP resistors.

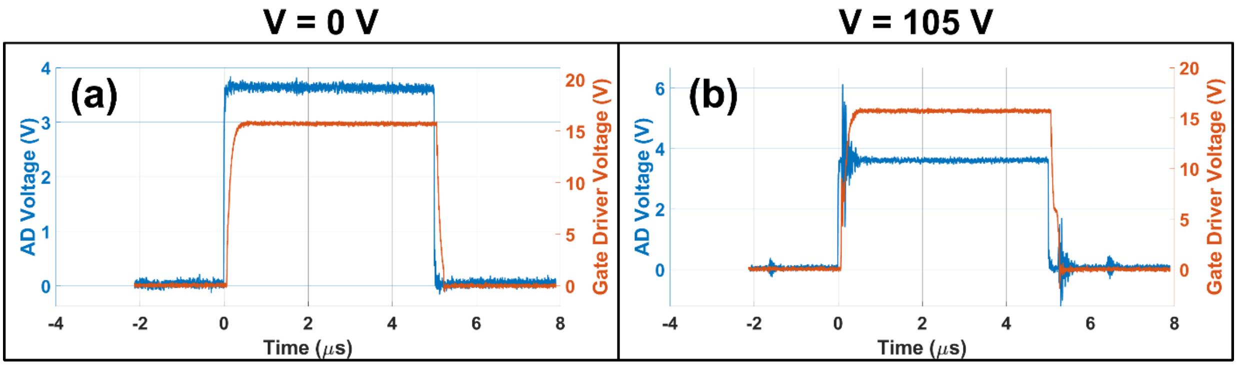

fig. S6. Electrical Signal Integrity

Electrical Signal Integrity -- illustrates the signal integrity from the Analog Discovery (AD) digital 3.3 V input and the 15 V gate driver output. (a) shows the Analog Discovery 3.3 V input signal and 15 V gate driver output signal with an applied 5 µs, V = 0 V driving voltage pulse (no resistor current flows in this test case). (b) shows the Analog Discovery 3.3 V input signal and 15 V gate driver output signal with an applied 5 µs, V = 105 V driving voltage pulse. Fast switching of large currents (2-3 A) leads to electromagnetic interference on the digital 3.3 V input signal which can be seen in (b). The gate driver output signal has a system rise and fall time of 48 ns and 93 ns respectively to reach the power MOSFET 5 V threshold voltage to fully turn on. As shown in (b), the gate driver and bypass capacitors effectively eliminate large signal transients at the signal rise and fall due to electromagnetic interference seen on the digital input signal. As such, the developed PCB shown in figure S5 can be used to generate 0-300 V, > 200 ns voltage pulses to drive TMP resistors.

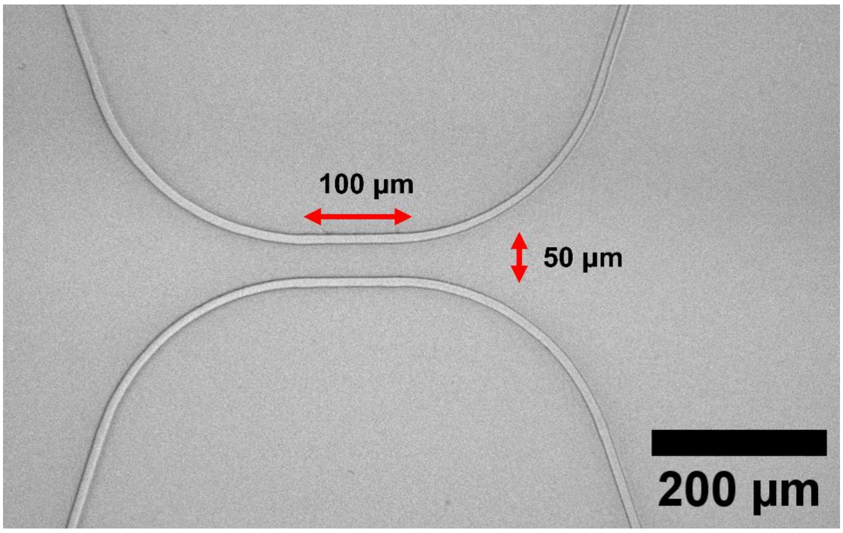

fig. S7. 50 x 100 µm Resistor. Illustrates the lower resolution bound of femtosecond laser fabrication of thermal micro-pump resistors.

table S1. Bill of Materials Summary. Summarizes the bill of materials for PCB fabrication, electrical components, and stroboscopic optical components used in this study. PCB electrical components are supplied from Mouser and listed in *Mouser PCB Bill of Materials.xls* and the PCB KiCad layout file used to order from OSH Park is provided in *4 Resistor Control KiCad OSH Park Board.kicad_pcb*. We note that the system can be made lower cost than presented here by the following substitutions: (1) using a custom transmitted light microscope instead of a pre-build reflective light microscope built from

**PCB Fabrication**				
Part	Quantity	Part Number	Cost ($)	Vendor
High Power PCB	3	n/a	151.80	OSH Park
PCB Parts – see attached bill of materials file	n/a	n/a	140.35	Mouser

**Electrical Components**				
Part	Quantity	Part Number	Cost ($)	Vendor
300V/5.6A DC Power Supply	1	Sorensen XG300-5.6	715.00	Ametek
60V/5A DC Power Supply	1	n/a	84.99	Amazon
Analog Discovery 2	1	410-321	279.00	Digilent
FTO Coated Glass	5	735183-5EA	88.20	Sigma-Aldrich

**Stroboscopic Components**				
Part	Quantity	Part Number	Cost ($)	Vendor
Basler CMOS Camera	1	a2A1920-160umBAS	359.00	Basler
Pulsed LED Light Source	1	HPLS-36DD18B-KIT	2,400.00	Lightspeed Technologies Inc.
White LED	1	LEDH-5500	included	Lightspeed Technologies Inc.
Red LED	1	LEDH-630	included	Lightspeed Technologies Inc.
Blue LED	1	LEDH-470	34.00	Lightspeed Technologies Inc.
C/CS Mount Collimator	1	OH36-24X11	135.00	Lightspeed Technologies Inc.
Reflective Light Microscope	1	Axiotron ZS-19	1,500.00	Zeiss
5X Objective	1	M-5X	125.00	Newport

extension tubes, 45° cube mirror, and an objective lens; and (2) using a high power Cree LED (Mouser, CXB3590-0000-000N0YBD56Q) for transmitted light instead of the HPLS-36DD18B pulsed light source.

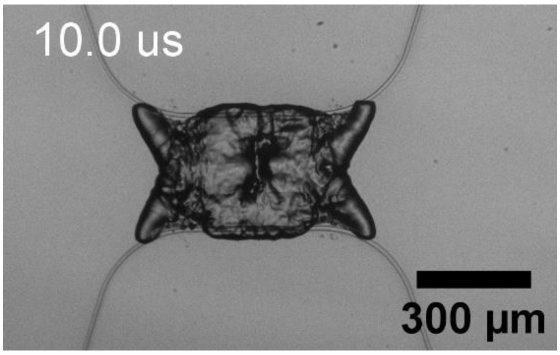

movie S1. Stroboscopic imaging of a femtosecond laser fabricated resistor in water, no channel. Stroboscopic high speed imaging of a 44.77 Ω, 300 x 700 μm, 250 μm fillet, 8 Ω/sq FTO femtosecond laser fabricated resistor with a 100V, 5 us pulse firing pulse in water with no channel and imaged at 2 Mfps.

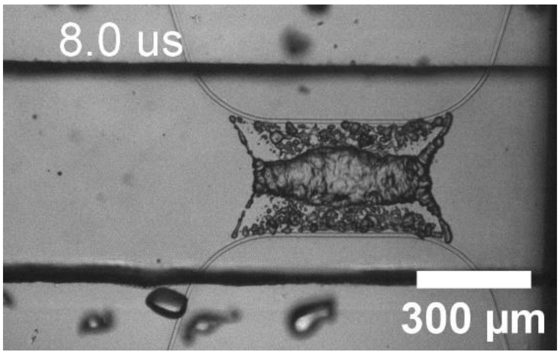

movie S2. Stroboscopic imaging of a femtosecond laser fabricated resistor in water, with channel. Stroboscopic high speed imaging of a 45.99 Ω, 300 x 700 μm, 250 μm fillet, 8 Ω/sq FTO femtosecond laser fabricated resistor with a 105V, 5 us pulse firing pulse in water in a 515 x 305 μm^2 channel and imaged at 2 Mfps.

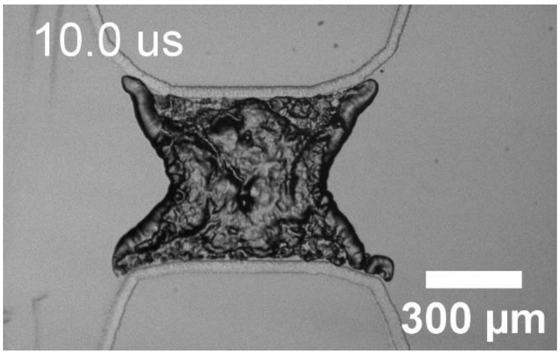

movie S3. Stroboscopic imaging of a Trotec fiber laser fabricated resistor in water, no channel. Stroboscopic high speed imaging of a 38.59 Ω, 500 x 700 μm, 250 μm fillet, 8 Ω/sq FTO

Trotec fiber laser fabricated resistor with a 150V, 5 us pulse firing pulse in water with no channel and imaged at 2 Mfps.

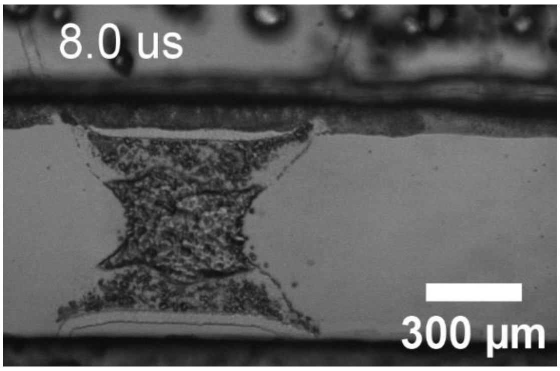

movie S4. Stroboscopic imaging of a Trotec fiber laser fabricated resistor in water, with channel. Stroboscopic high speed imaging of a 38.33 Ω, 500 x 700 µm, 250 µm fillet, 8 Ω/sq FTO Trotec fiber laser fabricated resistor with a 150V, 5 us pulse firing pulse in water in a 740 x 340 μm^2 channel and imaged at 2 Mfps.

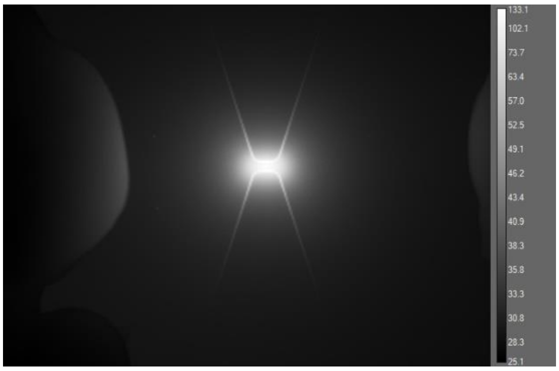

movie S5. FLIR Thermography Imaging of a Resistor Heating Pulse. Thermography video of a 45.50 Ω, 300 x 700 µm, 250 µm fillet, 8 Ω/sq FTO resistor with a 6.5V, 20 s pulse firing pulse in air.

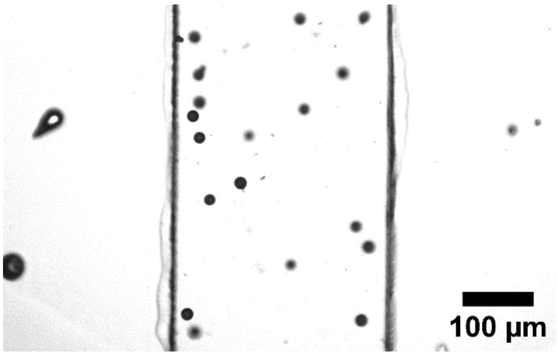

movie S6. Particle Tracking Video Example. Video of 27-32 µm diameter particle movement during pumping.
