# Supplementary material for: Rapid Fabrication of Low-Cost Thermal Bubble-Driven Micro-Pumps
Source: Micromachines (Basel). 2022 Sep 29;13(10):1634. doi: 10.3390/mi13101634 (PMC9610814; doi:10.3390/mi13101634)
Supplement: Supplementary file 1 [file micromachines-13-01634-s001.zip › controller GUI S1.zip!/Python Control Scripts/Stroboscopic Imaging User Manual.pdf]

Stroboscopic Imaging User Manual

Initializing / Setup:

1. Be sure emergency off button is engaged
2. Connect power cable and BNC cable to the light source

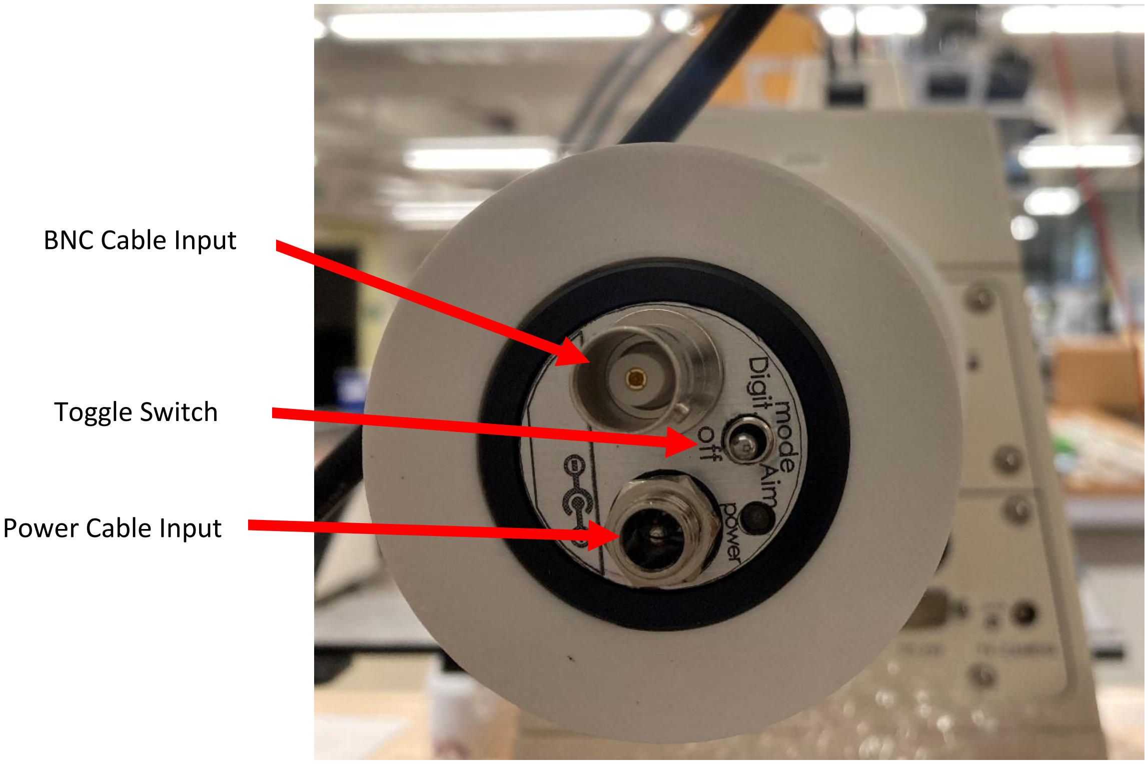

3. Turn the toggle switch on light source to “aim” mode for pulsed firing
4. Turn on the Sorensen XG-300-5.6 power supply on the bottom shelf and press the main power enable
5. Turn on the Eventek DC power supply on the top shelf

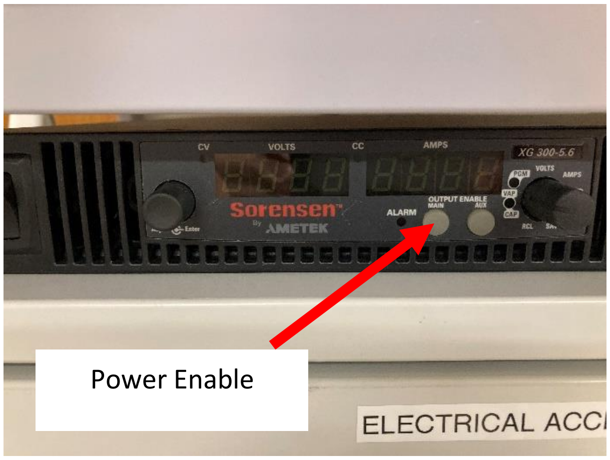

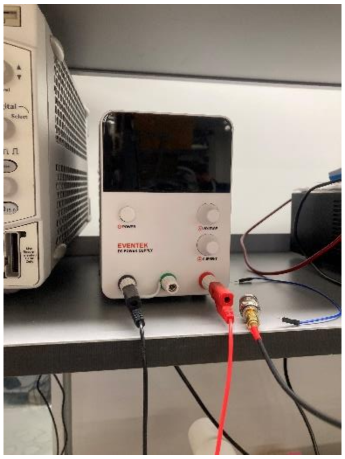

6. Plug the USB cable into the USB 3.0 port of the computer. It has the connections for both the analog discovery control system and the basler camera.

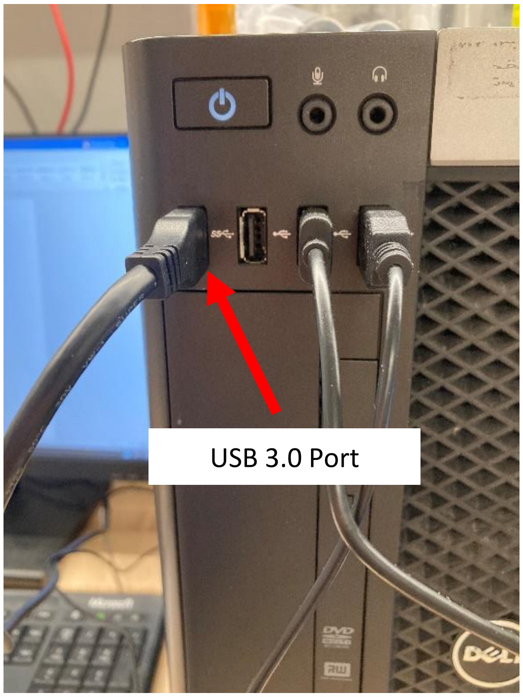

7. Turn off and back on the power on the back USB strip for the analog discovery and camera systems. This allows the system to hard restart and not error.

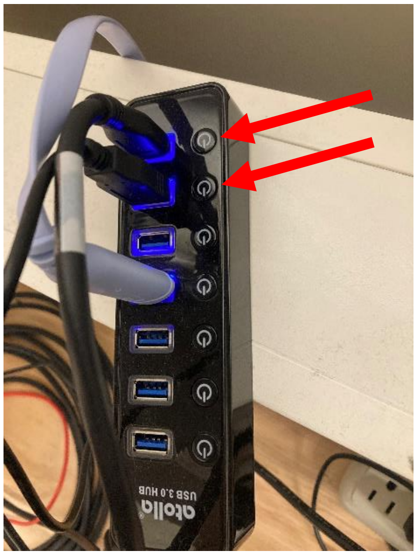

Collecting Data:

1. Open the GUI for the stroboscopic imaging setup. Can find all GUI's here: C:\Users\MacLab\Documents\Python Control Scripts
2. **Be sure emergency off is pressed and the voltage reads 0.0 V on both the PCB voltage display and the power supply. Could turn off the power supply when handling the wires.** Connect the wires to the resistor to close the circuit.

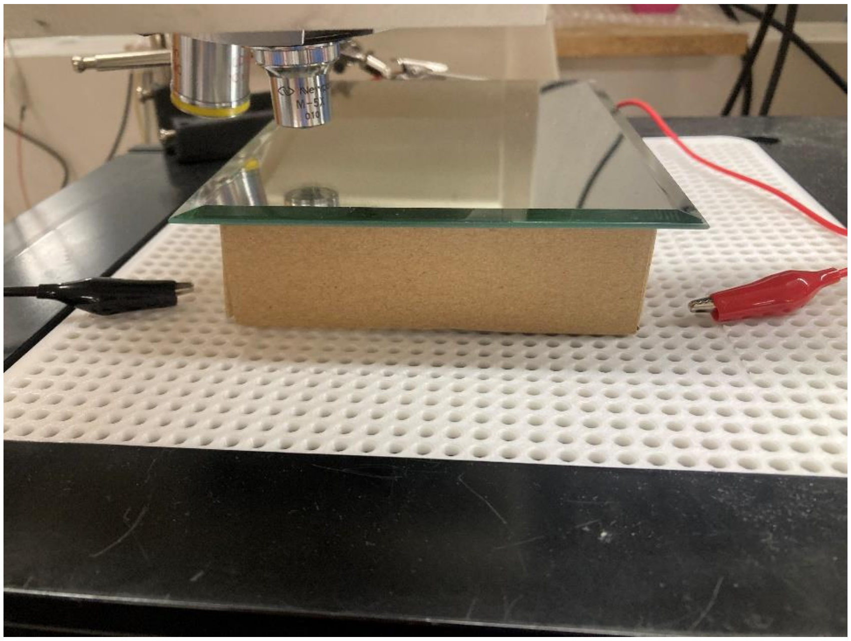

3. The following example is for the "Manual_Strobing_GUI"

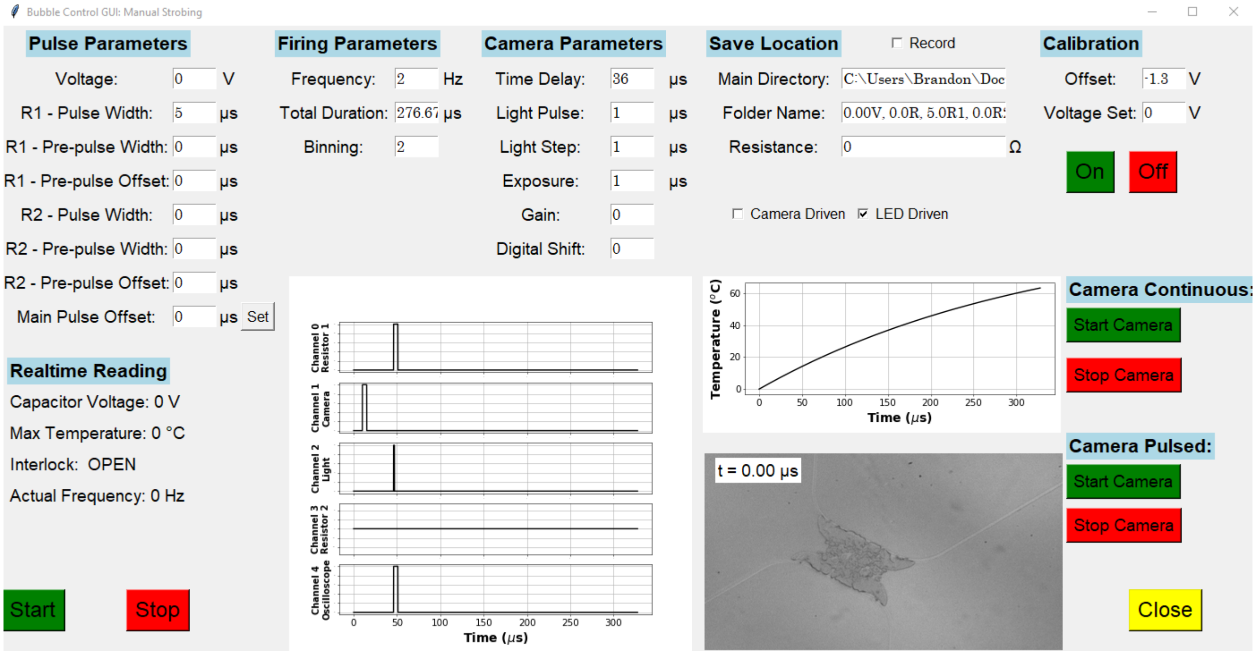

4. Camera pulsed: lets you visualize through the microscope using the pulsed light source with the set parameters in the “camera parameters” section. Will need to always “stop camera” before hitting “start”
5. Set the “camera parameters” section to the desired imaging settings. Exposure should be 1 us when the light pulse / light step is 1 us or less (as when strobing) but the exposure can be set higher to 3 us (as when collecting particle information).
6. Set the “main directory” to which the files should be saved. Folder name automatically populates when hitting the “set” button. Record the resistance value if needed.
7. Check the “calibration” voltage to make sure the right output voltage on the power supply is outputted.
8. Set the “pulse parameters” and “firing parameters” to desired values.
9. Press “start” to begin a test with imaging feedback. If you want to record data, select “record” before hitting “start”.
10. Definition of all parameters for reference:
    a. Voltage: what voltage the power supply should be set to
    b. R1 – Pulse Width: sets the pulse width of the resistor firing pulse
    c. R1 – Pre-pulse Width: sets the pre-pulse width of the resistor firing pulse
    d. R1 – Pre-pulse Offset: sets the pre-pulse offset between the firing pulse and the pre-pulse
    e. Main Pulse Offset: sets the offset between the first firing pulse and the second for two resistor configurations
    f. Frequency: sets the firing frequency of the resistor (also the imaging frame rate since an image is taken after every pulse)
    g. Total Duration: sets the total duration to record
    h. Binning: sets the value of pixels to bin. Usually set at 2
    i. Time Delay: sets the camera timing offset which is required for it to get ready before recording an image. Not needed to adjust
    j. Light Pulse: sets the duration of the light pulse
    k. Light Step: sets the time interval to record at. Should match the light pulse used
    l. Exposure: sets the basler camera exposure time
    m. Gain: sets the basler camera gain
    n. Digital Shift: sets the basler camera digital shift
    o. Main Directory: sets the saving directory for the results
    p. Folder Name: sets the folder name for the results
    q. Resistance: sets the resistance value measured for the resistors
    r. Offset: sets the voltage offset to use to tune the output voltage
    s. Voltage Set: calibrates the voltage to XX volts to estimate the required offset
11. **If system ever exits abnormally or you exit it, hit the emergency stop and double check that the voltage display reading on both the power supply and next to the PCB read 0.0 V**
12. **Hit the emergency off whenever not using the system**

Shutdown Procedure:

1. **<u>Make sure the emergency off is pressed</u>**
2. Turn off Sorensen XG-300-5.6 power supply
3. Turn off Eventek DC power supply
4. Unplug BNC and power cable to light source
5. Turn toggle switch to middle position on light source (“off”)
6. Unplug the USB connector from the USB 3.0 computer port
7. Double check that the system is in a safe state before leaving
